# Epigenetic Regulation of S100A9 and S100A12 Expression in Monocyte-Macrophage System in Hyperglycemic Conditions

**DOI:** 10.3389/fimmu.2020.01071

**Published:** 2020-06-02

**Authors:** Dieuwertje M. Mossel, Kondaiah Moganti, Vladimir Riabov, Christel Weiss, Stefan Kopf, Julio Cordero, Gergana Dobreva, Marianne G. Rots, Harald Klüter, Martin C. Harmsen, Julia Kzhyshkowska

**Affiliations:** ^1^Medical Faculty Mannheim, Institute of Transfusion Medicine and Immunology, Heidelberg University, Mannheim, Germany; ^2^Department of Dermatology, University of Münster, Münster, Germany; ^3^Department of Medical Statistics, Biomathematics and Information Processing, Medical Faculty Mannheim, Heidelberg University, Mannheim, Germany; ^4^Department of Medicine I: Endocrinology and Clinical Chemistry, University Hospital Heidelberg, Heidelberg, Germany; ^5^Anatomy and Developmental Biology, CBTM, Medical Faculty Mannheim, Heidelberg University, Mannheim, Germany; ^6^Department Pathology and Medical Biology, University Medical Centre Groningen, University of Groningen, Groningen, Netherlands; ^7^German Red Cross Blood Service Baden-Württemberg – Hessen, Mannheim, Germany

**Keywords:** diabetes mellitus, inflammation, macrophage, epigenetic, histone code, metabolic memory

## Abstract

The number of diabetic patients in Europe and world-wide is growing. Diabetes confers a 2-fold higher risk for vascular disease. Lack of insulin production (Type 1 diabetes, T1D) or lack of insulin responsiveness (Type 2 diabetes, T2D) causes systemic metabolic changes such as hyperglycemia (HG) which contribute to the pathology of diabetes. Monocytes and macrophages are key innate immune cells that control inflammatory reactions associated with diabetic vascular complications. Inflammatory programming of macrophages is regulated and maintained by epigenetic mechanisms, in particular histone modifications. The aim of our study was to identify the epigenetic mechanisms involved in the hyperglycemia-mediated macrophage activation. Using Affymetrix microarray profiling and RT-qPCR we identified that hyperglycemia increased the expression of *S100A9* and *S100A12* in primary human macrophages. Expression of *S100A12* was sustained after glucose levels were normalized. Glucose augmented the response of macrophages to Toll-like receptor (TLR)-ligands Palmatic acid (PA) and Lipopolysaccharide (LPS) i.e., pro-inflammatory stimulation. The abundance of activating histone Histone 3 Lysine 4 methylation marks (H3K4me1, H3K4me3) and general acetylation on histone 3 (AceH3) with the promoters of these genes was analyzed by chromatin immunoprecipitation. Hyperglycemia increased acetylation of histones bound to the promoters of *S100A9* and *S100A12* in M1 macrophages. In contrast, hyperglycemia caused a reduction in total H3 which correlated with the increased expression of both S100 genes. The inhibition of histone methyltransferases SET domain-containing protein (SET)7/9 and SET and MYND domain-containing protein (SMYD)3 showed that these specifically regulated *S100A12* expression. We conclude that hyperglycemia upregulates expression of *S100A9, S100A12* via epigenetic regulation and induces an activating histone code on the respective gene promoters in M1 macrophages. Mechanistically, this regulation relies on action of histone methyltransferases SMYD3 and SET7/9. The results define an important role for epigenetic regulation in macrophage mediated inflammation in diabetic conditions.

## Introduction

Diabetes confers an about 2-fold higher risk for cardiovascular disease (1, 2). Type 1 diabetes (T1D) is an autoimmune disease in which the immune system destroys pancreatic beta cells and renders patients dependent on insulin administration. Type 2 diabetes (T2D) is associated with insulin resistance i.e., the inability of tissue cells to respond to insulin. Obesity is a strong predictor for T2D, while T2D itself increases risks for cardiovascular disease and cancer. Both in T1D and T2D the chronic exposure to increased glucose serum levels (hyperglycemia) causes pathophysiological changes that are largely of an inflammatory nature (3, 4). Macrophages are important in homeostasis of metabolism of tissues as well as whole body metabolism whereas on the other side, the intrinsic metabolism of the cell shapes its activation state (5, 6). Macrophages respond to their environment with either pro-inflammatory (M1) or anti-inflammatory (M2) fashion and can be polarized in both directions, defined as “macrophage plasticity.” In diabetic retinopathy activated resident macrophages i.e., microglia, produce inflammatory, and neurotoxic mediators that that disrupt vascular integrity and function (7). Macrophage recruitment and activation contribute to diabetic nephropathy (8) and diabetic neuropathy (9). The inability to switch phenotype from pro-inflammatory to anti-inflammatory macrophages, has been hypothesized to mediate delayed wound healing seen in diabetic patients (10).

Metabolic memory referes to the beneficial effects i.e., reduced incidence and progression of diabetic complications, of intensive glycemic control seen after return to normal therapy during the Diabetes Control and Complications Trial (DCCT) (11). Post-prandial hyperglycemia and spikes in glucose levels may not reflect fasting plasma glucose and the commonly used serum marker for diabetes Hemoglobine A1c (HbA1c) (12, 13). Therefore, transient periods of hyperglycemia can also cause in diabetic patients cellular changes that do not alter upon normalization of blood glucose levels (14, 15). Epigenetic modifications have been postulated to confer hyperglycemic memory in target cells involved in vascular dysfunction including endothelial cells (ECs), vascular smooth muscle cells (VSMCs), and renal mesangial cells (MCs) (16). Chemical modifications at histone tails that surround gene promotors can inhibit or stimulate gene expression. Most-studied modifications are methylation and acetylation on Histone 3 (H3) that mainly occur on the side chains of lysines (K) and arginines residues (17).

Since S100 genes do not contain CpG islands, and were not identified in methylation screening assays on diabetic samples, we decided to focus on histone modifications which generally preceed DNA methylation in epigenetic reprogramming. Histone code also is essential for macrophage programming in inflammatory conditions related to infections (18, 19). Monocytes can respond in different ways upon restimulation. Chromatin modifications discriminate opposing functional programs for either enhanced (training) or decreased (tolerance) cytokine production, depending on the type of stimuli encountered (20–22). In a diabetic (micro)environment, glucose and its byproducts i.e., Advance glycation endproducts (AGEs) cause epigenetic changes in the bone marrow. This causes a diabetic preconditioning of monocytes and macrophages (23, 24). Blood monocytes of diabetic patients who had joined the DCCT showed increased H3K9Ac on gene promoters related to NF-κB inflammatory pathways. High H3K9ac levels correlated with HbA1c and progression of retinopathy or nephropathy after 10 year of follow-up in patients with type 1 diabetes (25). Also, several histone modification on the promoters and enhancers of inflammatory regulators in macrophages are related to the progression of atherosclerosis (26). Histone modifications are written and removed by histone modifying enzymes. Histone methyltransferases responsible for H3K4me are the histone methyltransferase Mixed Lineage Leukemia (MLL) family, SET1A, SET1B, SET7/9, SMYD, and PRDI-BF1 and RIZ homology domain containing (PRDM)9. Differences are that SET7 exhibits monomethylation activity whereas SMYD3, MLL1/2, and MLL3/4 trimethylate H3K4 (27). SET7 has been shown to be sensor for hyperglycemic changes in EC (28) whereas MLL has been found to be important in macrophages differentiation (29, 30).

Recenlty we have performed a systematic analysis of the effect of hyperglycemia on the trascriptional program of differentially activated human primary macrophage subtypes. We showed that hyperglycemia upregulates expression of several members of the S100 protein family. The highest effect of hyperglycemia was gene expression of *S100A9* and *S100A12*, in particular in pro-inflammatory M1 macrophages which are maturated with MCSF and simultaneously stimulated with IFNγ, and for *S100A8* in M0 macrophages, maturated without additional stimulation (Supplementary Table 1). The original array data for all differentially activated genes is accessible at NCBI GEO database accession GSE86298 and will be published elswhere.

Highest levels of S100A9 are expressed in neutrophils and monocytes, while expression of S100A12 is more restricted to neutrophils (31, 32). However, S100 proteins are also produced and function in other cell types like keratinocytes, fibroblasts, epithelial, and endothelial cells (33, 34). S100A9 and S100A12 are produced during inflammatory conditions, and their biological effects depend on different activation states of the producing cells, concentration as well as the composition of the local milieu (35). Both proteins activate cells via RAGE (36, 37) and S100A9 activates TLR4 (38) but also regulates macrophage function via CD68 (39). Macrophage migration is promoted by S100A9 via Extracellular Matrix Metalloproteinase (ECM) Inducer EMMPRIN (CD147) (40). S100A9 is regulated by MMPs (41) but also blocks MMP degradation of the ECM (42). S100A9 appears to control the oxidative potential of the NADPH complex, S100A8/A9 binding to cell receptors induces signal transduction through NF-κB pathways (40, 43, 44). Besides formation of homomultimers, S100A9 may dimerize with S100A8, or form S100A8/A9 tetramers called calprotectin (45). Pro-inflammatory activity of S100A9 can be restricted by formation of the calcium-induced (S100A8/S100A9)_2_ tetramer that can not bind TLR4/MD2, thus preventing undesirable systemic inflammatory effects (46). Genome-wide transcriptional profiling of nerve stumps in the sciatic nerve axotomy model in rats identified that S100A8 and S100A9 are key factors that initiate the early inflammatory program in injured peripheral nerves (47). Ccalprotectin is an acute phase protein and detects already minimal inflammation levels and is suggested as biomarker in (chronic) inflammatory diseases (48, 49).

Expression levels of *S100A8, S100A9* (50, 51) and circulating levels (52, 53) of S100A12 (ENRAGE) and soluble receptor for (R)AGE (54, 55) positively correlate with diabetes pathology. Serum levels of S100A9 and calprotectin were higher in T1D patients compared to healthy controls (56), and correlated with the progression of diabetic retinoptahy in T2D patients (57), but also with insulin resistance/type 2 diabetes, metabolic risk score, and fat cell size caused by obesity (58). S100 proteins are major RAGE ligands and inflammation through RAGE is thought to be central target in diabetic complications as well as diabetes induced cancer (59).

Thus, taking into account that S100 proteins are essential regulators of inflammation and their elevated levels are associated with diabetes, in this study we focused on the mechanism of regulation of S100 gene expression under hyperglycemic conditions in macrophages as key innate immune cells that contribute to both inititation and progression of diabetes and its complications. By analysis of S100 gene expression we tested the hypothesis that hyperglycemia in diabetic patients induces long-term activation through epigenetic mechanisms similar to trained immunity (24, 60) in primary human macrophages.

## Materials and Methods

### Peripheral Blood Mononuclear Cell (PBMC) of Diabetic Patients

Frozen PBMC samples of diabetic patients seen at the University Hospital Heidelberg, Germany were used in the study. All studies were approved by the ethics and review committee of Medical Faculty Heidelberg, University of Heidelberg (ethic-vote-number S-383/2016; clinical trial number NCT03022721). For gene expression analysis by RT-qPCR, PBMCs from healthy controls (*n* = 21), Prediabetic individuals (*n* = 19), T1D (*n* = 19), and T2D (*n* = 21) patients were obtained (clinical data are presented in Supplementary Table 2). Pre-diabetes was defined based on increased fasting glucose between 100 and 125 mg/dl or an impaired glucose tolerance—with elevated blood glucose levels between 140 and 199 mg/dl after intake of 75 g glucose. Within the group of T1D patients 75% of the subjects suffered from neuropathy, 37.5% from retinopathy and 17.6% from nephropathy. Also, patients with the group with T2D diabetes suffered from polyneuropathy (76.2%) and/or nephropathy (52.4%) and showed albuminuria. For analysis by flow cytometry, PBMCs from T2D patients with microvascular complications (*n* = 11) compared to healthy controls (*n* = 4) were used of which the clinical data are presented in Supplementary Table 4. Samples were processed as descibed in detail below.

### Monocyte Isolation and Generation of Macrophages

Human monocytes were isolated from buffy coats from individual donors as described (61) with modifications. Buffy coats were provided by the German Red Cross Blood Service Baden-Württemberg – Hessen. Buffy coats were obtained from healthy donors after informed consent. Selection of monocytes occurred through selection by anti-CD14 antibodies and magnetic activated cell sorting (MACS) (Milteny Biotech, US). The obtained monocytes were cultured at 1 × 10^6^ cell/ml in customized serum free medium (SFM) with 5 mM (normal glucose) and 25 mM (high glucose) glucose (Life Technology, Germany) at a concentration of 1 × 10^6^ cells/ml, supplemented with cytokines (see below) in the presence of 7.5% CO_2_ for the time periods up to 6 days without medium change. For ChIP experiments 20 × 10^6^ cells were seeded in 100 mm cell culture dish and for RNA-isolations 3 × 10^6^ in 6-well-plates without additional coatings. Cells were incubated with cytokines derived from PeproTech (Germany) in the presence of 7.5% CO_2_ for 6 days. 5 ng/ml Macrophage colony-stimulating factor (MCSF) and 100 ng/ml interferon-gamma (IFNγ) was used to induce M1 macrophage polarization and 5 ng/ml MCSF with 10 ng/ml IL-4 to induce M2 macrophage polarization. M0 macrophages were but MCSF not additionally stimulated (ns). For all reagents used, identifiers are listed in a Key Resource Table (Supplementary Table 5).

### RNA Isolation and cDNA Synthesis

Cells were lysed in TRK lysis buffer and RNA was isolated using E.Z.N.A. Total RNA kit (Omega Bio-tek, USA) according to the manufacturer's instructions. The concentration of isolated RNA was determined with a Tecan Infinite® 200. cDNA synthesis was performed using Fermentas RevertAid cDNA synthesis Kit (Thermo Scientific, US) with oligo-dT primers according to the manufacturer's instructions. The obtained cDNA was diluted 1:10 with double distilled water and 1 μl was used for PCR.

### Flow Cytometry

Frozen PBMCs from diabetic patients and healthy controls, were thawed and plated in RPMI medium supplemented with 10% fetal calf serum (FCS) and 1% penicillin, streptomycin and 5 mM of glucose. Cells were incubated overnight at 37°C, with the diabetic group consisting of patients with severe complications, consisting of nephropathy and additional patients with nephropathy and cardiovascular disease. The next day, the cells were harvested and washed with Phosphate buffered saline (PBS). Fixable viability dye (FVD, Thermofisher) was added to all unstained, IgG control and stained Fluorescence-activated cell sorting (FACs) tubes and incubated for 30 min at 4°C protected from light. Cells were washed twice with FACS Buffer (PBS, 0.4% BSA, 0.02% NaN3). Ten microliter FcR Blocking Reagent (Miltenyi Biotec) was added to all tubes and incubated for 5 min at RT. Antibodies (Table 1) were added to stained tube and incubated 20 min at 4°C in the dark. Cells were washed twice with cellwash, resuspended cells in PBS and fixed with 3.5% Formaldehyde while vortexing. After 15 min at RT in the dark, cells were washed with PBS and resuspended in 0.1% Saponin (Roth) and left on ice for 15 min. Cells were centrifuged, resuspended in 0.1% Saponin. Ten microliter FcR block was added and incubated for 5 min on ice. For intracellular staining antibodies or isotype controls for the critical colors were added to the respective tubes and incubated for 30 min on ice. Cells were washed twice with 0.1% Saponin, resuspended in FACS Buffer and analyzed by BD FACS Canto II. Antibodies for the following markers were used: Human leukocyte antigen-DR (HLA)-DR, CD3, CD19, CD56, CD16, and CD14 (Biolegend). Names of antibody clones are provided in the Key Resource Table (Supplementary Table 5). Cells were selected that were positive for HLA-DR. Cells positive for CD3, CD19, and CD56 were excluded. Using a scatter plot of CD16 vs. CD14 monocyte population were separated into classical (CD14+CD16–), non-classical with low CD14 expression (CD14–CD16+) and intermediate (CD14+CD16+) monocytes. These populations were analyzed for the expression S100A9 and S100A12.

**Table 1:** Antibodies used for flow cytometry.

**Marker**	**Conjugate**	**Control isotype-matched ab**	**Volume per assay (μl)**
CD16	APC	Na	2.5
CD3	FITC	Na	1
CD19	FITC	Na	1
CD56	FITC	Na	1
CD14	PerCPCy5.5	Na	1
HLA-DR	PE Cy7	Na	0.5
S100A9	PE	IgG1, κ	0.5
S100A12	AF405	IgG2b	5

*Na, not applicable*.

### Gene Expression Analysis

Primers and probes were obtained from Eurofins (Germany). Dual-labeled probes were used containing 6-carboxyfluorescein (FAM) on the 5′ end and a Black Hole Quencher-1 (BHQ1) at the 3′ end of the sequence. Primers for 18S ribosomal RNA (*18S rRNA)* were designed (Table 2). Primer sequences are shown from the 5′ end to 3′ end direction. Taqman ready to use human primers for *S100A9* (Hs00610058_m1), *S100A12* (Hs00942835_g1) were obtained from Thermo Scientific (US). For endogenous control *18S rRNA* was used. Use of 18S was validated compared to other housekeeping genes (data not shown).

**Table 2:** Primers used for *18SRNA*.

**Gene**	**Primer**	**Sequence (written 5'-3')**
18S rRNA	Forward	CCATTCGAACGTCTGCCCTAT
18S rRNA	Reverse	TCACCCGTGGTCACCATG
18S rRNA	Probe	ACTTTCGATGGTAGTCGCCGTGCCT

### Chromatin Immunoprecipitation (ChIP)

ChIP was used to assess the relative abundance of activating histone marks at the promoter regions of the genes of interest. ChIP assays were performed with SimpleChIP® Enzymatic Chromatin IP Kit (Cell Signaling Technology, US) according to the manufacturer's protocol. To crosslink proteins to DNA, formaldehyde was added to the medium to a final concentration of 1% and incubated at RT for 10 min. Glycine was added for 5 min to neutralize unreacted formaldehyde. Media was removed and cells were washed twice with ice-cold PBS scraped and taken in 2 ml PBS + 10 μl (200x) PIC buffer provided in the kit. Cells were lysed and chromatin was digested within buffers provided. Digestion was done by micrococcal nuclease (2,000 units/μl) with an optimized ratio of 0.5 μl per 5 × 10^6^ cells harvested at 37° for 20 min. Chromatin was sonicated to obtain fragments of 150–900 base pairs. Digestion was analyzed by an 1% agarose gel. For immunoprecipitation digested chromatin of 5 × 10^6^ cells was diluted into ChIP buffer and and 2 μg of primary antibody H3K4me1 (Abcam, UK) H3K4me3and acetylated histone H3 (Merck Millipore, Germany) was used in a final volume of 0.5 ml and incubated at 4°C with rotation overnight. Normal rabbit immunoglobulin G (IgG) and total H3 (D2B12) wer used as controls for the IP. Immune complexes were captured using 30 μl of ChIP Grade Protein G Magnetic Beads provided. The chromatin was eluted in elution buffer provided and crosslinks were reversed by adding 6 μl 5 M NaCl and 2 μl Proteinase K and incubation for 2 h at 65°C. The DNA was purified using QIAquick PCR Purification Kit (Qiagen, Germany). The amount of precipitated genomic DNA concentration was measured with a Tecan Infinite® 200. Samples were subjected to RT-PCR using primers for different promoter regions of *S100A9, S100A12* (Tables 3, 4). One microliter of DNA was added to each well. PCR reactions included a 2% input sample and a well with no DNA to control for contamination. Signals obtained from each immunoprecipitation are expressed as a percent of total input chromatin. IP efficiency was calculated with the following equation: Percent Input = 2% x 2(C_T_ 2%Input Sample – C_T_ IP Sample). 3,000 bp upstream of the transcription start site (TSS), defined by SwitchGear genomics tool in the Epigenomebrowser.org, was used to scan for suitable ChIP primers.

**Table 3:** Primers used for ChIP on human *S100A9* promoter.

**Gene promoter**	**Promoter region**			**Primer**	**Sequence 5'-3'**
S100A9				F	GCCTGGTGCTAAGACTTTGG
	P1	−108	17	R	GCATGACAATGAAGCAGGGT
				Pr	AGCAGGCAGCATCCCTGCCT
				F	TGAGCTCTTCCCAACTTTCCA
	P2	−551	−420	R	CTCACACTGCTGAGATGCAC
				Pr	ACTGCCTAAGGTCACACAGACAGTCTG
				F	GCATTACCACACTGCTCACC
	P3	−1241	−1117	R	GAGCCACACAGAGTGTTTGC
				Pr	TGGCCCTTTGGCCCTGTCTC
				F	TCCGGGTGTCAGTTTCTTCA
	P4	−1715	−1544	R	TGCCTGGCTCTGTGATACTTA
				Pr	TGCAAGAGGGTTGCCCACCTCT
				F	GCTGTGTGCATAGGAGAAGG
	P5	−2722	−2546	R	TCTGGCTCTCAACACTTGCT
				Pr	TGCCTCTGTCCAACAATTGGCTGTAGA

**Table 4:** Primers used for ChIP on human *S100A12* promoter.

**Gene promoter**	**Promoter region**			**Primer**	**Sequence 5'-3'**
S10012				F	ACAGCCTGAGTGTCTTGTTT
	P1	−83	56	R	ACTGATCCTCTGCTCCAGTG
				Pr	ACCTCCTCCTAAGTCGTTCTGGGATGC
				F	CCCACACCTGTGAAGATAAGC
	P2	335	497	R	CCCACCCAGGTTGGTTTCTA
				Pr	ACCAATCTCACAACTTGCCCACAAGGA
				F	AGGGCTAAGATGAAGCCTGA
	P3	909	1,045	R	ACCACCTAAGAACCCATCCA
				Pr	TGCCCTTCACCACTGCTGGC
				F	GGGATGCAGGAGAACAGACA
	P4	1,562	1,734	R	GGCAGTTTGTGTTTGGTGGT
				Pr	TGCTCCCACTGCCTGGTGCT
				F	CAATCAAGGCCATGCCAGAA
	P5	3,517	3,627	R	CACATGGATCGGAGAGACAGA
				Pr	TGTGCCCACCGACCTCTCTGG

### Viability Assay

Alamar blue solution (Life technologies, Germany) 10% was added to the medium and the cells and the cells were incubated in the presence of 7.5% CO_2_ at 37°C for 3 h. Fluorescence was measured in triplicates at 590 nm read by Tecan Infinite® 200. Fluorescence of pure AlamarBlue was used as a negative control.

### Inhibition of Histone Modifying Enzymes

Primary human derived macrophages were obtained as previously described. The regulatory effect of HMTs on transcription of *S100A9* and *S100A12* was analyzed using specific inhibitors. M1 macrophages which were cultured in the presence of MCSF and IFN-γ under normal and high glucose conditions were treated with inhibitors for SET7, SMYD3, and Mixed Lineage Leukemia (MLL) histone methyltransferases (HMTs). (R)-PFI-2 hydrochloride, a substrate-competitive inhibitor which occupies the substrate peptide binding groove of SET7 (62) and EPZ031686 inhibitor for SMYD3 (63) were derived from MedChem Express (US). WD Repeat Domain 5 (WDR5) 0103 inhibitor of MLL which disrupts WDR5 interaction with MLL and inhibits MLL core complex methyltransferase activity (64) was obtained from Bio-techne (US). The inhibitors dissolved were in DMSO and corresponding concentrations of DMSO were used as controls. Cells were treated at the indicated concentrations from the time of isolation on up to 6 days followed by RNA isolation.

### Immunofluorescence Staining

Monocyte-derived macrophages were stimulated with MCSF and INF-γ and cultured on cover slips (Neolab, germany) for 6 days under normal and high glucose conditions. Cells were fixed using 2% paraformaldehyde (PFA) in PBS for 10 min and washed with 0.5% TritonX-100 in PBS for 15 min to permeabilize. Intracellular structures were fixed with 4% PFA 10 min again. Cells were washed three times with PBS and stained for SET7/SET9 (Cell Signaling, US). DRAQ5 (Life Technologies, Germany) was used for nuclear staining. Expression and localization was analyzed using the Leica TCS SP8 confocal laser scanning microscope. Analysis of fluorescence intensity and the nucleus size in pixels (regions of interest defined by DRAQ5 staining) were performed using Fiji software (imagej.net).

### Statistics

All statistical calculation have been done with the statistical software SAS, release 9.4 (SAS institute Inc., Cary, North Carolina, USA). For qualitative factors, absolute, and relative frequencies are given. Quantitative variables are presented by their mean value and standard error. In order to compare the mean values of two independent samples, a 2 sample *t*-test has been used. For data not normally distributed Mann Whitney *U*-test has been used instead. In order to compare more than two samples, a one way ANOVA or a Kruskal-Wallis-Test has been performed, as appropriate. In the case of a statistically significant test result, *post-hoc* tests according to Scheffe or Dunn's test have been applied, respectively. For the comparison of the mean values of two paired samples (i.e., days 1 and day 6), a two paired *t*-test has been used. In order to evaluate simultaneously the impact of two factors on a quantitative outcome, a two way ANOVA has been done. If necessary, an ANOVA with repeated measurements have been applied (i.e., for donors which have been measured several times).

Correlation coefficients according to Spearman have been used in order to quantify the degree of association. In general, the result of a statistical test has been considered as significant for *p* < 0.05 (^*^P < 0.05; ^**^P < 0.01; ^***^P < 0.001 and ^****^P < 0.0001).

## Results

### Hyperglycemia Increases the Expression of S100 Genes During Monocyte/Macrophage Differentiation Under IFNγ Stimulation

**Figure 1:**
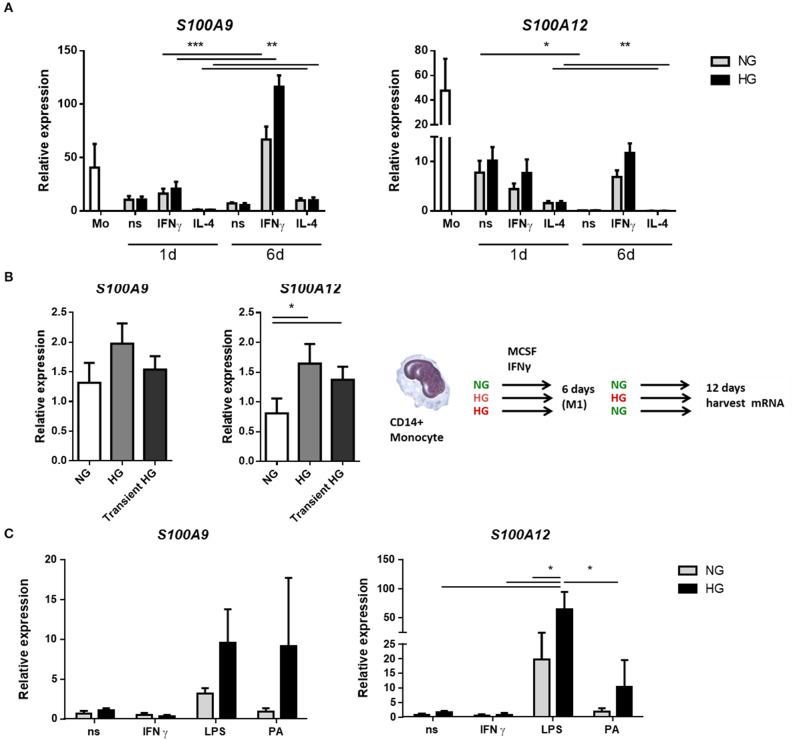
Hyperglycemia sustains the mRNA expression of S100 proteins during monocyte/macrophage differentiation. **(A)** RT-PCR analysis of mRNA expression *S100A9* and *S100A12* in M0_ns_, M1_IFNγ_, and M2_IL−4_ macrophages cultured for 24 h or 6 days under normal (NG, 5 mM) and high glucose (HG, 25 mM) conditions, *n* = 10. **(B)** M1 macrophages were generated in HG medium for 6 days, after which cultures were reverted to NG for another 6 days (transient HG). Controls were maintained under, respectively, NG or HG the whole experiment, *n* = 7. **(C)** M0 macrophages cultured for 6 days in NG or HG conditions and stimulated for 16 h with TLR-ligands. Data present mean ± SEM normalized to *18srRNA* expression levels, *n* = 8. Statistical analysis was performed using two-way ANOVA with Scheffe's test **(A)** one-way **(B)** and Kruskal-Wallis with Dunn's test **(C)**.

We determined the expression of *S100A9* and *S100A12* at day 1 and day 6 of differentiation from monocytes into M1 resp. M2 macrophages. It is known that *S100A8 and S100A9* mRNA levels decline during monocyte differentiation into macrophage (65). Compared to macrophages, monocytes presented the greatest mean expression levels of both *S100A9* and *S100A12* regardless of glucose. Both genes declined during monocytes to macrophage maturation and *S100A12* was downregulated much stronger than *S100A9* (360-fold and 5.4-fold, respectively, for ns, NG after 6 days) (Figure 1A). The effect of the stimulator on gene expression was present from day 1 whereas glucose influenced gene expression only from day 6 on. After 6 days, the highest levels of *S100A9* and *S100A12* were found in M1 compared to M0 and M2 macrophages (*P* < 0.0001 for both genes). In addition, 9 out of 10 donors showed increased *S100A9* expression, up to 4.9-fold for individual donors, and 8 out of 10 donors showed increased *S100A12* expression, up to 3-fold for individual donors after 6 days in M1 macrophages cultured under high glucose conditions. We also analyzed protein levels of S100A9 and S100A12 in monocytes and macrophages from healthy donors by Western blot (Supplementary Figure 1). Similar as observed for mRNA expression, we observed on protein level that S100A9 and S100A12 proteins were more abundantly present in monocytes compared to matured macrophages (not shown).

For the analysis of hyperglycemic memory in M1 macrophages, after 6 days, when the monocytes had differentiated into macrophages, the medium was changed from NG to NG, from HG to HG and from HG back to NG (transient hyperglycemia, Figure 1B) while MCSF and IFNγ was maintained. mRNA expression levels were analyzed after 12 days. Higher expression of *S100A9* was observed in HG-HG compared to NG-NG only (*P* = 0.0386 by paired *t*-test). The increase was found in 5 out of 7 donors with highest increase for individual donors being 2.8-fold (Figure 1B). When the medium was reversed to normal glucose levels, expression of *S100A9* and *S100A12* was increased in 4 of 7 donors compared to macrophages that were maintained in NG medium. For *S100A12*, higher expression was observed in HG-HG compared to NG-NG conditions. Expression increased in 5 out of 7 analyzed donors with highest levels for individual donors being 8.6-fold (Figure 1B). When the medium was reversed to normal glucose, higher *S100A12* expression was observed compared to cells that were in NG medium continuously. The increase was found in 6 out of 7 donors with highest levels of for individual donors being 5.7-fold (Figure 1B). Thus, the observed differences at day 6 (Figure 1A) remain present at day 12.

Next, we investigated the effect of secondary pro-inflammatory stimuli on glucose conditioned M0 macrophages by measuring gene expression of *S100A9* and *S100A12* (Figure 1C). Monocytes were cultured in normal and high glucose conditions and after 6 days these were challenged overnight with TLR-ligands, Palmitic acid (PA) is a saturated fatty acid with a role in atherogenesis and T2D (66). We observed that LPS induced expression of both *S100A9* and *S100A12* compared to non-stimulated controls and IFNγ stimulated cells. This only reached significance for *S100A12*. We observed that high glucose dramatically increased the expression levels of *S100A9* in response to PA (9.9-fold). Expression of *S100A12* was upregulated by glucose in LPS stimulated cells (5.4-fold). Also other TLR-ligands/inflammatory stimulators FSL1 and Pam3CK4 were used and *IL-1*β, *IL-6*, and *IL-10* gene expression was measured. It was found that IL-1β and IL-6 expression also increased under LPS in the HG cells. There was no difference in expression between NG and HG cultured cells when stimulated with FSL1 and Pam3CK4 (data not shown).

### S100A9 and S100A12 Expression in Monocytes of Diabetic Patients

**Figure 2:**
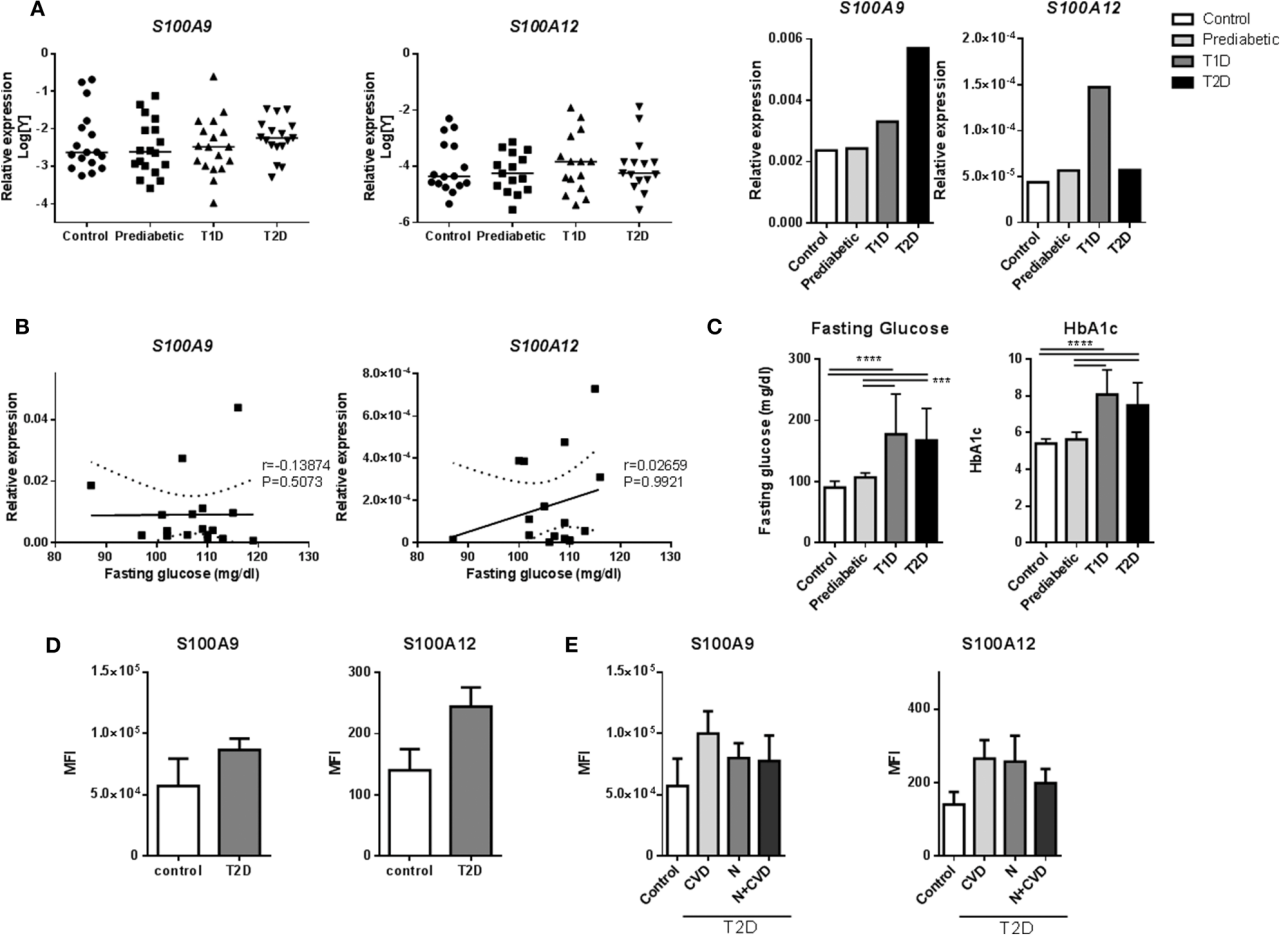
S100A9 and S100A12 expression in monocytes of diabetic patients. **(A)** RT-PCR analysis of S100A9 and S100A12 expression in PBMCs from prediabetic individuals, diabetic patients and healthy controls normalized to CD14 expression. Data in bargraph present medians. Statistical differences between groups was tested with Kruskal-Wallis-test. **(B)** Correlation between S100A9 and S100A12 expression and fasting glucose in pre-diabetic individuals. Number of XY Pairs = 16. Graphs show 95% confidence band of best-fit line. Spearman's correlation coefficient (r) and *p*-value are given in the graphs. **(C)** Fasting glucose and HbA1c levels in prediabetic individuals, diabetic patients and healthy controls. Control (*n* = 21), Prediabetic (*n* = 19), T1D (*n* = 19), and T2D (*n* = 21). (One-way ANOVA with Scheffé-*post-hoc* test). **(D)** S100A9 and S100A12 expression was analyzed by flow cytometry. Classical, intermediate and non-classical monocytes subsets from T2D patients with microvascular complications (*n* = 11) compared to healthy controls (*n* = 4). Cells positive for HLA-DR were selected and all cells expressing CD3, CD19, CD56 were excluded. Monocytes were gated based on CD14+ and CD16+ expression and frequencies of parent (HLA-DR population) are plotted. Mann-Whitney-*U*-test. **(E)** S100A9 and S100A12 expression in T2D patients with Cardiovascular disease (CVD), nephropathy (N), or both, compared to healthy controls. Statistical analysis by one-way ANOVA, data present mean +SEM.

We assessed whether *S100A9* and *S100A12* genes are higher expressed and therefore relevant in monocytes from prediabetic and diabetic patients (Supplementary Table 2). Expression of *S100A9* and *S100A12* in diabetic patients was not different compared to controls in our dataset (Figures 2A,B). Interestingly, *S100A9* and *S100A12* expression were tightly correlated in T1D, T2D and healthy controls (*P* = < 0.0001 for all three) but not in prediabetic individuals (Supplementary Table 3). Similarly, we observed *in vitro* that the tight correlation between *S100A9* and *S100A12* was lost in M1 macrophages cultured in high glucose conditions compared to NG (data not shown). FG or HbA1c levels in prediabetic individuals were not as high as seen in diabetic patients (Figure 2C). In prediabetics, monocytic inflammatory gene expression did not correlate with fasting glucose levels (Figure 2B). Some individuals with high expression had high fasting blood glucose levels (Figure 2B). Also, we did not observe a correlation between gene expression levels and HbA1c levels, BMI or weight (data not shown).

Also, intracellular levels of S100A9 and S100A12 protein were assessed in monocytes from diabetic patients with microvascular complications (Supplementary Table 4). Monocytes are classified into three subtypes based on CD14+ and CD16+ expression, which differ in function and phenotype (67). By FACS (representative FACS plot for gating strategies; Supplementary Figure 2), the frequencies of these monocyte populations did not differ between controls and T2D patients (Supplementary Figure 3). S100A9 and S100A12 expression was determined in all CD14+ positive cells and within the different subgroups according microvascular complications. There was a trend for higher proportion (frequency) of S100 positive cells in HLA-DR, lin- subset (cells positive for HLA-DR with CD3, CD19, and CD56 positive cells excluded) for S100A9 (35 ± 16 vs. 28 ± 11%) as well as for S100A12 (28 ± 11 vs. 24 ± 12%) in T2D samples compared to healthy controls (data not shown). Also, the MFI, the average intensity of protein expression, of S100A9 in T2D was 1.5-fold higher than healthy controls (86,485 ± 30,754 compared to 57,027 ± 44,782 units) as well as for S100A12, 1.7-fold increase compared to healthy controls (244 ± 103 compared to 140 ± 69 units) (Figure 2D). S100 protein expression in patients with CVD compared to controls or patients with nephropathy, did not differ in this dataset (Figure 2E). Further, no correlation of S100A9, S100A12 protein expression was found with HbA1c and other metabolic factors i.e., BMI, HDL or fasting glucose (data not shown).

### Local Chromatin Structure of S100A9 and S00A12

**Figure 3:**
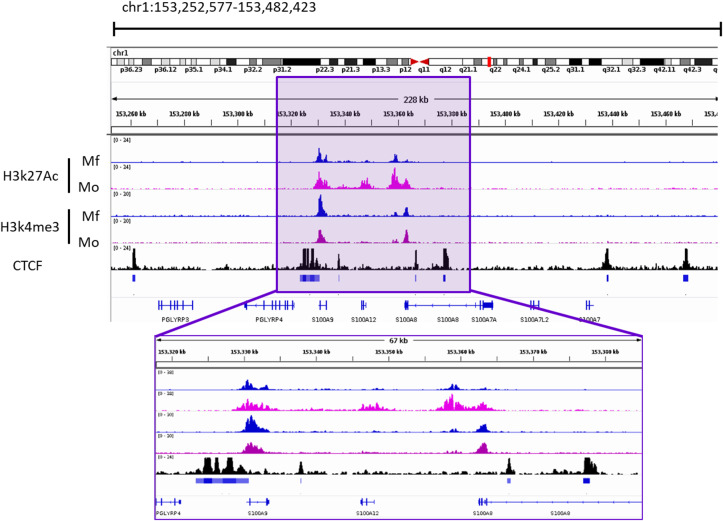
Occupancy of H3k27ac and H3k4me3 from Macrophages (Mf) and Monocytes (Mo) From GSE109440 dataset. CTCF-ChIP-seq enrichment around *S100A9* and *S100A12* genes. Data taken from Mo GSM1003508. Square highlights the enriched area of histone marks and CTCF In the S100A9 and S100A12 genomic locus. IGV Snapshot in the lower part is a zoom in of the area of interest.

Given the proximity of the two genes and their possible co-regulation, we analyzed a publicly available dataset on local chromatin structure by ChIP-seq from primary human monocytes (68). We found enrichment of H3K27ac and H3K4me3 on TSS < 1kb of both genes. Additionally, CTCF-ChIP-seq data showed marked enrichment in CTCF sites 1kb upstream of S100A9 and 31kb downstream of S100A12, overlapping with the second intron of S100A8. Together, these data indicate an active transcription area in S100A9 and S100A12 locus which correlates with the expression of these genes. One smaller CTCF site was found in between the two genes. More pronounced CTCF boundaries were found to include S100A8 gene as well. In the same study, differential H3K27Ace occupancy was found in monocytes compared to macrophages at the promoter of S100A12. This was not the case for S100A9, also pointing toward independent transcriptional activity regulation (Figure 3).

### Hyperglycemia Contributes to Association of Activating Histone Marks at S100A9 and S100A12 Promoters

**Figure 4:**
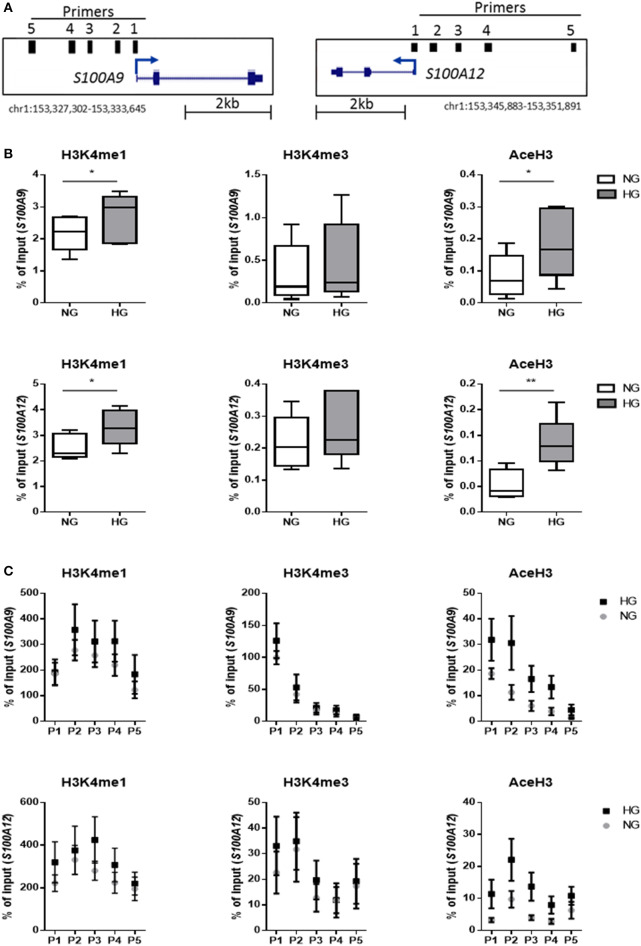
Histone code at promoter regions of *S100A9* and *S100A12*. **(A)** DNA regions in the promoter of *S100A9* and *S100A12*. **(B)** Level of histone modifications in *S100A9* and *S100A12* promoter regions, average of 5 regions, Min to Max plot. Rabbit IgG was used as a negative control for the pull-down. Histone modifications are presented as percent of input DNA and normalized to total H3 (by D2B12 antibody). Single comparisons between NG-HG made by students paired *t*-test, *n* = 5. A 2 sample *t*-test has been used to compare mean values. **(C)** level of histone modifications in 5 different regions of the *S100A9* and *S100A12* promoter, mean + SEM. ANOVA for repeated measurements have been performed for region (P1-P5) and glucose.

We investigated whether hyperglycemia affects the histone codes on the promoters of *S100A9* and *S100A12* genes. Using five individual donors, we analyzed the abundance of epigenetic marks in five regions of S100 gene promoters in M1 macrophages (Figure 4A). Hyperglycemia-induced effect on the activating histone modifications H3K4me1, H3K4me3, and AceH3 on promoters of both *S100A9* and *S100A12* was similar (Figure 4B). H3K4me1 on the promoters of both *S100A9* and *S100A12* genes had increased 1.3-fold for both genes (*P* = 0.0.0160 and *P* = 0.0196, respectively), while association of AceH3 had increased at both *S100A9* and *S100A12* promoters (2.4 and 2.5-fold, respectively, *P* = 0.0129 and 0.0054 respectively, Figure 4B). The region with highest level of H3K4me3 at the *S100A9* promoter was at the transcription start site (TSS) whereas general acetylation has highest association at 1,200 and 1,600 bp upstream from the TSS (Figure 4C).

**Figure 5:**
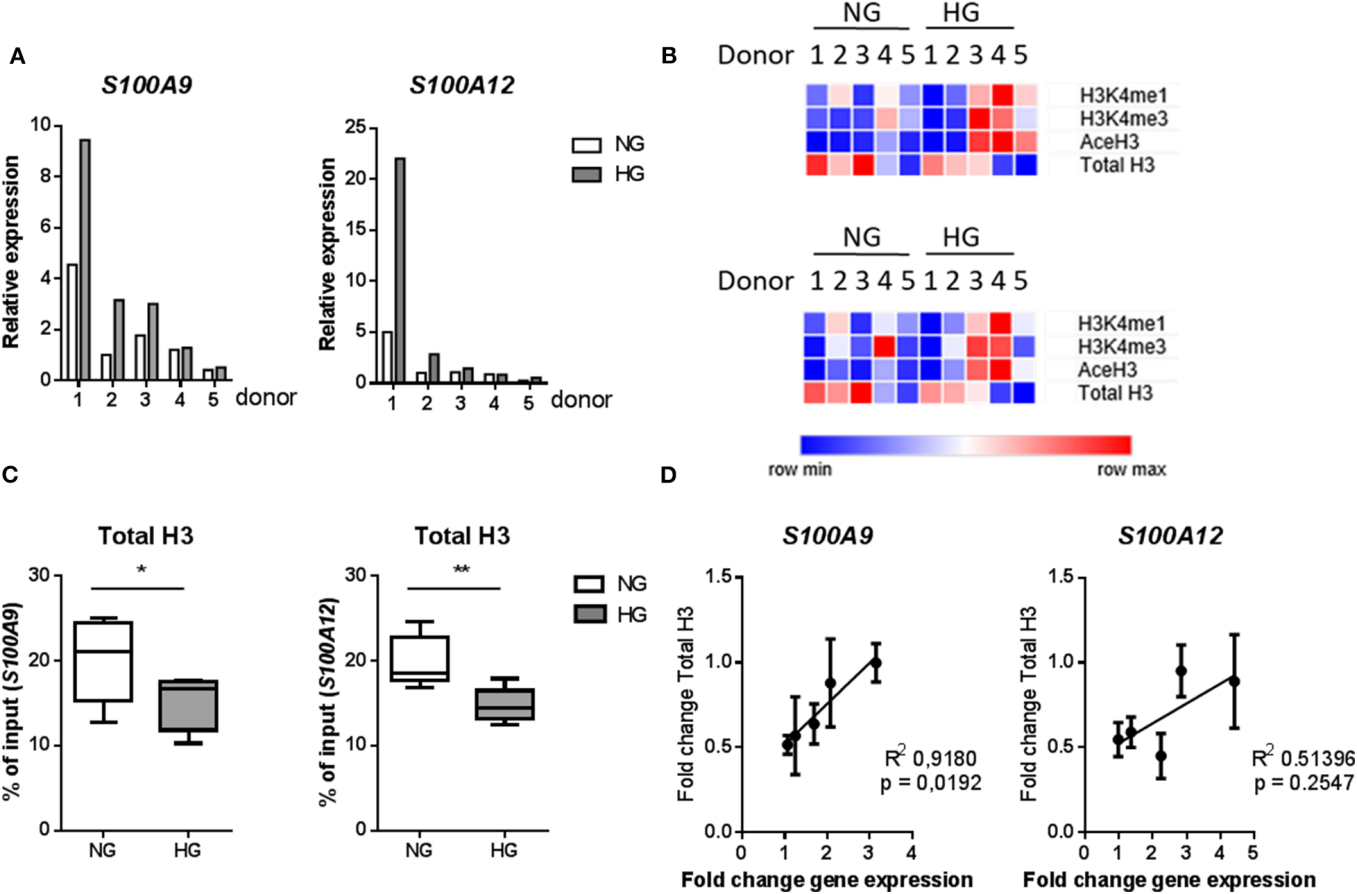
Changes in histones code correlate with gene transcription. **(A)** mRNA levels of *S100A9* and *S100A12* in M1 macrophages cultured for 6 under NG or HG conditions. **(B)** Heatmap of histone modifications at *S100A9* (top) and *S100A12* (bottom) promoter of the 5 individual donors. **(C)** Total H3 levels (by D2B12 antibody). Comparisons made using a students paired *t*-test. Min to max, *n* = 5. **(D)** Linear regression analysis of change in gene expression with total H3, the correlation coefficient was calculated by H3-mean and fold-change-values with *n* = 5.

We further examined whether these specific histone modifications correlated with gene transcription. Gene expression was increased under hyperglycemic conditions in M1 macrophages in 4 out of 5 donors for both *S100A9* and *S100A12* (Figure 5A). Donors 1 and 2 showed the highest level of gene expression and increase in expression upon culture in HG conditions (Figure 5A). However, the large change in gene expression did not come along with pronounced epigenetic changes (Figure 5B). Whereas, donor 3, 4, and 5 (to a lesser extent) which show only small increases in gene expression, respond with large increases of H3K4me1, H3K4me3, and AceH3 (Figure 5B). Therefore, fold change increase in gene expression correlated negatively with increase in level of histone modifications. On the other side, we observed that total H3, i.e., nucleosome density, was reduced under hyperglycemic conditions in all donors (Figures 5B,C). Also, stronger induction of gene expression (donor 1 and 2) by HG then was associated with the least reduction in total H3, hence a positive correlation of fold change in total H3 with fold change in gene expression (Figure 5D). Changes in levels of H3 for the five individual primers e.g., specific promoter regions, were also examined. For *S100A9* P2 and P4 region as well as P1 region within *S100A12* promoter, which is the nearest region to the transcription start site, an almost linear correlation was observed between fold change increases in H3 and fold change increase in gene expression (Supplementary Figure 4).

### Effect of Transient Hyperglycemia on the Presence of Activating Histone Marks at Promoters of S100A9 and S10012 Genes

**Figure 6:**
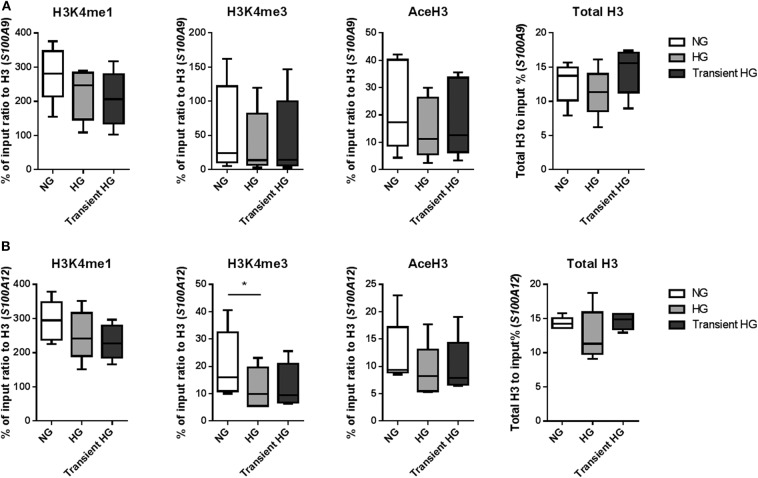
Modifications of histones at promoter regions in transient hyperglycemia. **(A)** M1 macrophages were generated in HG medium for 6 days, after which cultures were reverted to NG for another 6 days (transient HG). Controls were maintained under, respectively, NG or HG the whole experiment. Level of histone modifications at *S100A9* **(A)** and *S100A12* **(B)** promoter regions, average of 5 regions, Min to Max plot. Rabbit IgG was used as a negative control for the pull-down. Histone modifications are presented as percent of input DNA and normalized to total H3. ANOVAs with repeated measurements have been performed to evaluate differences between the three groups (HG, NG, Trans). Additional Scheffe *post-hoc* test for pairwise comparisons, *n* = 4.

Next, using four individual donors, we performed ChIP analysis on M1 macrophages were after 6 days high glucose medium reversed to normal glucose levels. Gene expression was increased in M1 macrophages in 3 out of 4 donors for *S100A9* and all donors for *S100A12* in both HG as well as transient hyperglycemia compared to NG. Transient hyperglycemia still presented 26 and 60% of the fold change increase induced by HG for *S100A9* and *S100A12*, respectively. By paired *t*-tests significant only, H3K4me3 as well as AceH3 on the promoter of both *S100A9* and *S100A12* were decreased in cell cultured continuously under HG conditions, unlike previously shown at day 6. H3K4me1 for *S100A9* and H3K4me3 for *S100A12* were sustained in transient hyperglycemia, significant by paired *t*-test only. Presence of activating histone marks negatively correlated with the increase in gene expression, similar to day 6 (data not shown). Changes in AceH3 and total H3 were reversed in transient HG whereas changes in H3K4 mono and trimethylation were similar between HG and transient HG (Figure 6). Changes in H3 correlated positively with fold change increases in gene expression for *S100A9* in transient hyperglycemia only (Supplementary Figure 5).

### Inhibition of SET7 Affects Both S100A9 and S100A12 Expression

**Figure 7:**
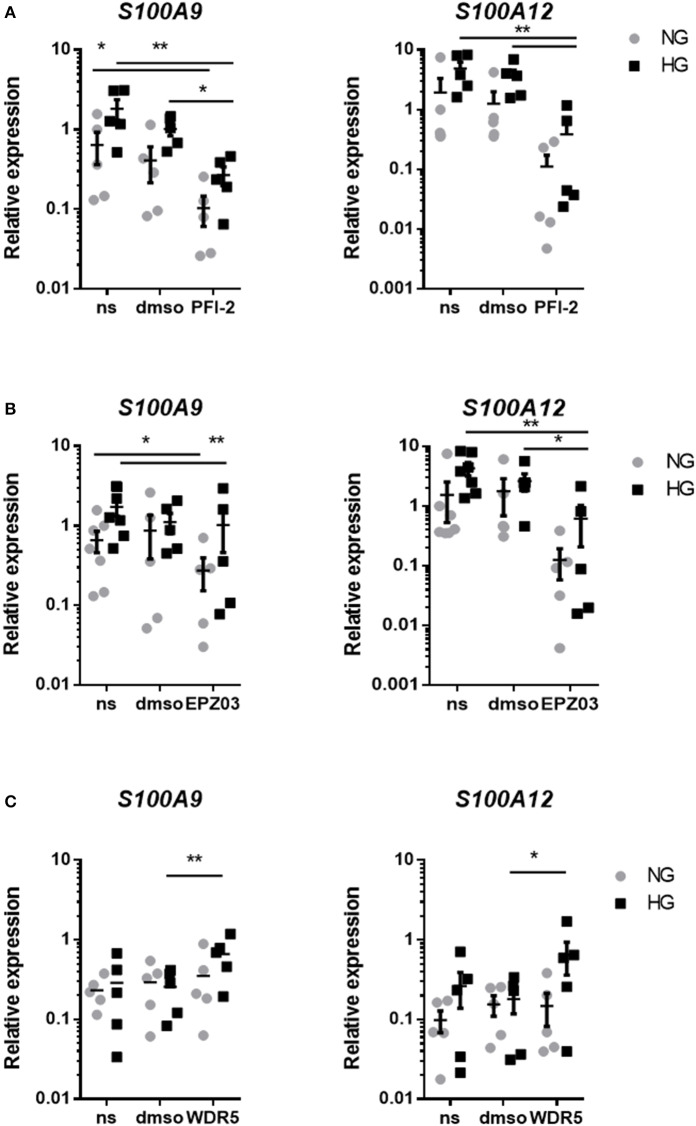
Regulation of *S100A9* and *S100A12* expression after inhibition of HMTs. RT-PCR analysis of the effect of treatment with PFI-2 hydrochloride inhibitor for SET7 10 μM concentrations corresponding dilutions of DMSO **(A)**, EPZ031686 inhibitor for 20 μM SMYD3 and corresponding to dilution of DMSO **(B)** or WDR5 0103 inhibition of MLL activity in 25 μM concentration and corresponding vehicle control **(C)**. Data present mean ± SEM normalized to *18S rRNA* levels. Two-way ANOVA have been performed including glucose (HG and NG) and stimulator (DMSO, PFI and ms) as two fixed factors. Further exploratory analyses for each gene and glucose according to Scheffé.

Our next aim was to identify which histone modifying enzymes mediated methylation in our cultured macrophages. The used inhibitors did not affect the viability of cells at the concentrations used, tested by Alamar blue (data not shown). Epigenetic modifying enzymes were inhibited in a dose-dependent fashion and optimal working concentration was determined (data not shown). By two-way ANOVA we determined how two factors i.e., HMT inhibition and glucose influence *S100A9* and *S100A12* expression. Interaction between those terms was not significant. In all three experiments expression levels of *S100A9* and *S100A12* were affected by glucose (indicating that the donors responded to hyperglycemia) as well as stimulatory factor compared to non-stimulated cells and solvent controls). We examined the relative contribution of methyltransferases on S100 gene upregulation by HG. Compared to the solvent control, inhibition of SET7 led to an 3.8-fold decrease in both NG and HG conditions for *S100A9* and 11-fold in NG and 9.4-fold decrease for *S100A12* in HG (Figure 7A). Compared to the solvent control, inhibition of SMYD3 downregulated expression of *S100A9* 3.2-fold in NG and 1.1-fold in HG conditions, compared to a 14-fold downregulation in NG and 3.4-fold in HG conditions for *S100A12* (Figure 7B). Therefore, cells grown in HG were more resistant to the effects of HMTs inhibitors. On the other side, inhibition of MLL activity increased *S100A9 and S100A12* expression (Figure 7C). Compared to its solvent control the increase was 1.2-fold in NG and 2.6-fold in HG conditions for *S100A9* compared to 1-fold in NG and 3.6-fold in HG conditions for *S100A12* indicating that WDR5 inhibition tends to synergistically increase expression together with glucose.

### Glucose Affects SET7 Expression and Localization in M1 Macrophages

**Figure 8:**
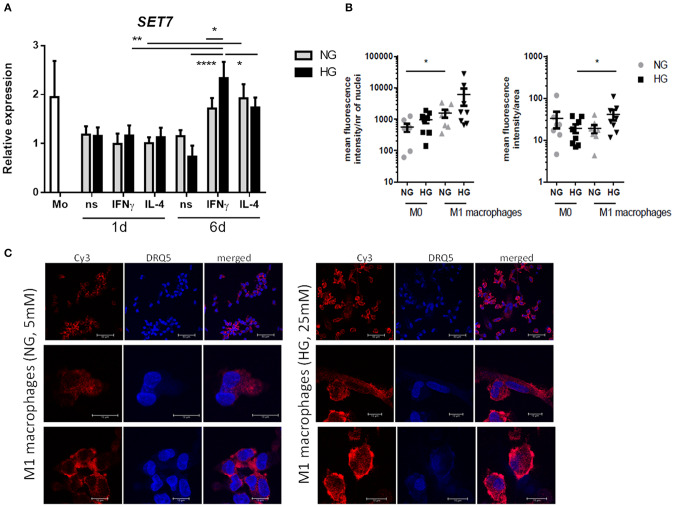
*SET7* expression and localization in primary human macrophages. **(A)** RT-PCR analysis of expression *SET7* in M0 _ns_, M1 _IFNγ_ and M2 _IL−4_ macrophages cultured for 24 h or 6 days cultured under NG and HG conditions. Data present mean ± SEM normalized to *18SrRNA* levels. Two-way ANOVA has been used for day 1 and 6 separately. Because of the interaction between day and stimulation, paired *t*-test has been used for comparison between the time-points **(B,C)** Immunofluorescence/confocal imaging of SET7 protein expression in M1 macrophages cultured for 6 days cultured under NG and HG conditions. Quantification of nuclear SET7 using fluorescene intensity/nucleus and intensity/area. Cells were fluorescent labeled with primary antibody against SET7 from Rabbit and secondary antibodies were Cy3 donkey anti-rabbit (red) and DRAQ5 (blue) for nuclei. Scale bars from top to bottom equal 50, 10 and 10μm, *n* = 8.

Because our results showed that only H3K4me1 correlated with gene transcription (*P* = 0.0093 and < 0.0001 for *S100A9* and *S100A12*, respectively, by linear regression analysis) and SET7 inhibition downregulated S100 gene expression, we further investigated SET7 during macrophage polarization and under hyperglycemic conditions. We observed that *SET7* expression did not change in response to glucose or cytokines after 1 day of macrophage polarization (Figure 8A, Supplementary Figure 6). After maturation of monocytes to macrophages (day 6), compared to M0 macrophages, SET7 expression had increased in 4 out of 6 donors for M1 and all donors for M2 (*P* = 0.0007 for M1 and 0.0053 for M2 compared to M0) (Figure 8A, Supplementary Figure 6). M1 macrophages stronger increased expression of *SET7* at days 6 compared to day 1, under HG than in NG conditions (*P* = 0.0071 and 0.1203). For M2 macrophages this increase was only near significant and not affected by culture in glucose (*P* = 0.0495 and 0.0452). Because SET7 translocates to the nucleus in endothelial cells in response to high glucose (69) the localization of SET7 was investigated in M0 and M1 macrophages by confocal microscopy in NG and HG conditions. Both M0 and M1 type macrophages expressed SET7 in their cytoplasm irrespective of glucose concentration (data not shown). Quantification of nuclear SET7 showed that the fluoresence intensity of the nuclei was was higher in M1 macophages compared to M0 macrophages (2.8-fold *P* = 0.0273 in NG, compared to 6.3-fold *P* = 0.0777 in HG) (Figure 8B). The intensity/area in M1 in HG was higher compared to M1 macrophages in NG conditions 2.2-fold, *P* = 0.0472 meaning that the fluorescent signal in the nucleus is stronger in glucose cultured cells (Figure 8B). Hyperglycemic culture also caused nuclear localization of SET7. A speckled intranuclear pattern was observed (Figure 8C) that was absent in normoglycemically cultured M1 macrophages.

## Discussion

Our hypothesis was that hyperglycemia induces metabolic memory together with M1 skewing which is caused by epigenetic switching. We focussed on *S100A9* and *S100A12* as we found the expression of these genes to be increased by hyperglycemia during monocyte/macrophage differentiation under IFNγ stimulation. For S100A12, the increase was maintained up to 6 days after reversal to normoglycemia. Hyperglycemic conditions indeed increased the association of activating histone marks at *S100A9* and *S100A12* promoters which correlated negatively with increase in gene expression but positively with fold change in total H3. *S100A9* and *S100A12* gene expression might be regulated through SET7 and SMYD3 whereas *SET7* expression and localization itself is affected by glucose in M1 macrophages.

*S100A9* tend to be higher expressed in T2D and *S100A12* in T1D patients. In patient samples, also other *in vivo* factors affect the outcome compared to the hypergycemic conditions in our *in vitro* assays (e.g., fatty acids, TLR ligands). Another important factor is the effect of the treatment the patients receive (70). FG or HbA1c levels were not as high as seen in diabetic patients and therefore might have affected the *S100A9* and *S100A12* expression in the prediabetic individuals. *S100A9* and *S100A12* expression were tightly correlated in monocytes from T1D, T2D, and healthy controls but not in prediabetic individuals. Also in lung diseases, the increased expression of S100A12 in acute respiratory distress syndrome suggested that S100A12 is more important in the onset of neutrophil influx compared to stages of chronic inflammation. Indeed in sputum of cystic fibrosis and chronic obstructive pulmonary disease patients, higher levels of S100A8/A9 were observed (71). Therefore, the regulation and ratio of the two different genes might provide more insight in the mechanism of inflammation.

Hyperglycemia-induced changes of H3K4me1, H3K4me3, and AceH3 was similar at promoters of *S100A9* and *S100A12* in M1 macrophages. As in RAW 264.7 macrophage-like cells stimulated with LPS, it has been shown that high and ongoing transcription was marked by either H3K79me2 or H3K36me3 and showed specific enrichment of motifs recognized by the NF-κB and IRF proteins (72). A reason that we did not observe strong association with transcription might be that H3K4me3 and transcriptional initiation are tightly linked but elongation of RNApII afterwards might occur through methyltransferases recognizing H3K36me3 (72, 73). AceH3 was highly increased by glucose the interrogated overall *S100A9* and *S100A12* promoter regions. In line with a more open chromatin state, less nucleosomes were cross-linked to DNA, as reflected by total H3 variants as recognized by our D2B12 antibody. Promotors then might be more accessible to transcription factors that are upregulated by IFNγ. For half of the donors, it was observed that elements gaining AceH3 starting from lower levels, in concert gain H3K4me1 whereas H3K4me3 remained relatively constant, as reported before (22). Bone-marrow-derived macrophages (BMDM) from T1D mice also show increased total HAT activity and decreased HDAC activity relative to control macrophages (74). In yeast, acetylation at specific residues that negatively correlate with increased transcription was mostly seen at H4 (75).

Nucleosomes hamper TFs from binding the DNA and therefore are general repressors of gene transcription (76). Therefore, the positive effect of nucleosome density in our study is not intuitive and indicates that the general rule of activating and repressing marks is not valid in exceptional cases or acts independently from each other. Similarly, in a study where nucleosome density on a specific promoter was manipulated by changing guanine-cytosine (GC) content, the lowest GC% did not correlate to highest output i.e., promoter activity (77). Nucleosomes are not bound 100% of the time but assemble and disassemble in equilibrium and occupancy varies genome wide. A reduction in nucleosome number therefore can increase the variability of relative occupancy since the histone pool is finite (78). Relative high occupancy together with high DNA accessibility has been described before (79). Nucleosome depletion also increased 15% but not affected expression of 75% genes in yeast switched to glucose medium. These could be genes that are already induced, or the transcriptional activators and repressors may be dominant in gene regulation (80). Activators of transcription are believed to act by recruitment of chromatin remodellers (81) which promote nucleosome removal (82). Chromatin remodeling complexes such as SWItch/Sucrose Non-Fermentable (SWI/SNF) complex accommodate single nucleosomes and their action is coupled to Adenosine triphosphate (ATP) hydrolysis which biases the spontaneous unwrapping of the DNA (77, 82, 83). One key player in macrophages DNA wrapping is high mobility group box1 (HMGB1). It resides in nucleus but is secreted after LPS/IFNγ stimulus reducing histone content and activating transcription (84). It was found to be sensitive to and activated by exposure to high glucose (85). Most likely the combination of different activating histone marks determines, together with a decrease of bound H3, the activity of the *S100A9* and *S100A12* promotors, and histone content critical in interpreting chromatin organization as it constitutes one layer of epigenetic regulation.

Hyperglycemia inhibited downregulation of S100 genes by both SET7 and SMYD3 inhibition. The effect was always stronger for *S100A12* compared to *S100A9* and cells grown in HG conditions seemed more resistant to the inhibitory effects. On the other side, WDR5, presenting the MLL complex activity, tend to synergistically increase expression in the presence of glucose. The key histone methyltransferase that is activated by hyperglycemia is SET7/9 writing H3K4me1. SET7 is involved in inflammatory signaling and found to be a co-activator of NF-κB in THP-1 cells as well as in macrophages from diabetic mice (86). SET7 expression was increased and H3K4me1 on NF-kB p65 promoter was associated with expression of NF-kB-dependent oxidant/inflammatory genes COX2 and iNOS in PBMCs form T2D patients (87). In our M0 and M2 macrophages, virtually no expression of *S100A9* or *S100A12* was present, whereas *SET7* is expressed at a modest higher rate. Indicating that the expression of S100 proteins associates with SET7 expression only in M1 macrophages. Quantitative data indicated SET7 relocalisation to the nucleus. We cannot exclude that increased methylation is a combined effect of several methyltransferases or decreased activity of demethylases, which should be examined in further studies.In this study we show that SMYD3 has effect on specifically *S100A12* promoter. SMYD3 however, despite existing literature does not methylate H3K4 but far more efficiently methylates H4K5 (88) and is mainly involved in regulation of transcription and signal transduction pathways promoting cancer development (89). SMYD3-mediated methylation of MAP3K2 promoted the activation of the Ras/Raf/MEK/ERK signaling module in cancer cell lines (90). Here, we show a new role of SMYD3 in regulating S100 gene expression under diabetic conditions.

Members of the MLL family show preferential methylation levels and this is according to their localization in the chromatin e.g., SET1A and B are found at promoter and preferential trimethylate whereas MLL3 and 4 localize at enhancer regions as is H3K4me1 (91). H3K4me1 at the promoter region is even suggested to induce transcriptional silencing and restrict H3K4me3 reading, in macrophages among other cell types, although it is not clear whether this is provoked by MLL3/4 or the remaining methylation after demethylase activity (91, 92). This could be an explanation for the negative correlation of H3K4me1 with increased gene transcription. The authors also observed that H3K4me1 for a group of acutely inducible genes, was mediated by MLL3/4 and loss of this HMT even promoted stimulus-dependent i.e., LPS induced gene expression without changes in H3K4me3 levels (92).

After normalized glucose levels, memorable changes of S100 genes were found for *S100A12*. After 12 days the epigenetic picture was different compared to 6 days. This could be either effect of medium change or chronic exposure to HG, which probably switches on a negative feedback mechanism that start to inactivate chromatin by a decrease in activating marks on the promoter of pro-inflammatory genes. H3K4me1 for *S100A9* and H3K4me3 for *S100A12* possibly mark metabolic memory. Change in AceH3 as well as total H3 were reversible and therefore seem more dynamic and responsive to metabolic changes. That the changes on gene expression level are not as fast as changes in epigenetic marks could be a consequence of stabilization of RNA.

LPS induced expression of *S100A9*, and even more of *S100A12*, compared to non-stimulated controls. We observed that high glucose dramatically increased the expression levels of S100A9 in response to PA and of S100A12 in LPS stimulated cells. Therefore, hyperglycemia augments stimulation with TLR-ligands and S100 proteins are sensitive to glucose conditioning. It has been shown before that high glucose induces a priming effect in macrophages and sensitizes cells toward inflammatory response (93, 94). It might be that due to the fact that glucose directly elevates the expression of TLRs (95). Another possible explanation would be that chromatin on S100 promoters is already opened and presence of secondary pro-inflammatory mediators dramatically induce the expression of these genes. Probably, both metabolic and epigenetic changes contribute to observed effects in this study. Firstly, since glucose metabolism determines immune cell activation and also training of monocytes via the AKT–mTOR–HIF-1a pathway (96) it had been hypothesized that high circulating levels of glucose could program immune cells toward an inflammatory phenotype through increased glucose utilization via glycolysis (24). However, we observed that culture in high glucose conditions did not change glucose uptake of M0 and M1 macrophages (data not shown), and it has been suggested before that increased glucose supply, i.e., increased uptake alone are not sufficient to drive inflammatory activation and atherosclerosis in myeloid cells (94, 97, 98). Second, several metabolic characteristics of M1 macrophages i.e., ROS, NO and succinate, are important demethylase inhibitors and inhibiting glycolysis or stimulating mitochondrial metabolism reduced the formation of HDAC inhibitor lactate (99) which links metabolism and AceH3 levels. Third, hyperglycemia-induced ROS and methylglyoxal production has shown to regulate expression of RAGE, S100A8, S100A12, and HMGB1 expression, which was normalized by overexpression of mitochondrial uncoupling protein 1, superoxide dismutase 2, or glyoxalase I. Loss of GLO1 mimicked the effect of high glucose whereas overexpression of GLO1 normalized the increased binding of NFκB p65 and activator protein 1 to the respective promoters (100), which might be mediated by SET7 (101). At last, overexpression S100A8 and S100A9 led to increased IL-10, whereas TN-α and IL-1β did not change (102). IL-10 mediates many anti-inflammatory effects in macrophages, but also has a role in metabolic programming; it inhibits glycolytic flux by inhibiting translocation of GLUT1 to the membrane in LPS-stimulated murine BMDMs (103).

Overall, an upregulation of S100 proteins by endogenous and diabetes-relevant ligands in hyperglycemic conditions together with memorable changes of S100 genes expression suggests that they can be important players in diabetes-related inflammation. Our results define an important role for epigenetic regulation in macrophage mediated inflammation in diabetic conditions. It remains to be studied if targeting epigenetic enzymes would correlate with reduction of diabetes severity *in vivo* in preclinical models. Individual differences in response to hyperglycemia and pro-inflammatory stimuli suggest that S100 proteins can be used to distinguish between responders and non-responders toward hyperglycemia indicating risks in diabetes patients.

## Data Availability Statement

All relevant data is contained within the article. Raw data supporting the conclusions of this article as well as relevant materials such as protocols are available upon request to interested researchers.

## Ethics Statement

All studies were approved by the ethics and review committee of Medical Faculty Heidelberg, University of Heidelberg (ethic-vote-number S-383/2016; clinical trial number NCT03022721).

## Author Contributions

DM, KM, and JK contributed to the conception and design of the study. KM and DM established the methods. DM performed the research and analysis. JC and GD performed bioinformatic analysis and interpretation. CW performed statistical analysis of the data. VR, JK, MH, and MR contributed to the analysis and interpretation of the results. DM wrote the first draft of the manuscript. Preparation of patient samples and patient details was done by SK. HK, VR, JC, JK, and MR contributed to manuscript revision. All authors read and approved the submitted version.

## Conflict of Interest

The authors declare that the research was conducted in the absence of any commercial or financial relationships that could be construed as a potential conflict of interest.

## Abbreviations

18S rRNA: 18S ribosomal RNA
AGEs: Advance glycation endproducts
ATP: Adenosine triphosphate
BHQ1: Black Hole Quencher-1
BMDM: Bone-marrow-derived macrophage
CD: Cluster of differentiation
ChIP: Chromatin immunoprecipitation
COPD: Chronic Obstructive Pulmonary Disease
DCCT: Diabetes Control and Complications Trial
DMSO: Dimethylsulfoxide
DNA: Deoxyribonucleic acid
DRAQ5: Deep Red Anthraquinone 5
EC: endothelial cell
ECM: Extracellular Matrix Metalloproteinase
EMMPRIN: Extracellular Matrix Metalloproteinase Inducer (CD147)
ERK1/2: Extracellular signal–regulated kinase
FACs: Fluorescence-activated cell sorting
FAM: 6-carboxyfluorescein
FCS: Fetal Calf Serum
FG: Fasting glucose
G6PD: glucose-6-phospate dehydrogenase
GAPDH: Glyceraldehyde 3-phosphate dehydrogenase
GC: Guanine-cytosine nucleotides
GLO1: Glyoxalase I
GLUT1: Glucose transporter 1
H3K4me: Histone 3 lysine 4 methylation
HAT: Histone acetyltransferases
HbA1c: Hemoglobine A1c
HDAC: Histone deacetylase
HG: Hyperglycemia
HLA-DR: Human leukocyte antigen-DR
HMGB1: High mobility group box1
HMT: Histone methyltransferases
IFNγ: Interferon gamma
IgG: Immunoglobulin G
IL: Interleukin
JNK: c-Jun N-terminal kinases
LPS: Lipopolysaccharide
MACS: Magnetic activated cell sorting
MAPK: Mitogen-activated protein kinase
MC: Mesangial cell
MCSF: Macrophage colony-stimulating factor
MLL: Mixed Lineage Leukemia
MMP: Matrix Metalloproteinase
mTOR: Mammalian target of rapamycin
NADH: Nicotinamide-adenine-dinucleotide hydride
NF-κB: Nuclear factor kappa-light-chain-enhancer of activated B cells
NO: Nitric oxide
PA: Palmatic Acid
PBMC: Peripheral blood mononuclear cell
PBS: Phosphate buffered saline
PFA: Paraformaldehyde
PPP: Pentose-phosphate pathway
PRDM: PRDI-BF1 and RIZ homology domain containing
RAGE: Receptor for AGE
RNA: Ribonucleic acid
ROS: Rective oxygen species
RPMI: Roswell Park Memorial Institute
RT: Room temperature
RT-qPCR: Quantitative reverse transcription PCR
SET7/9: SET domain-containing protein 7
SFM: Serum-Free Media
SMYD3: SET and MYND domain-containing protein 3
SOD2: Superoxide dismutase 2
SWI/SNF: SWItch/Sucrose Non-Fermentable
T1D: Type 1 diabetes
T2D: Type 2 diabetes
TF: Transcription factor
TLR: Toll like receptor
TSS: Transcription start site
UCP1: Mitochondrial uncoupling protein 1
VSMC: vascular smooth muscle cell
WDR5: WD Repeat Domain 5.
