# Supplementary material for: Epigenetic Regulation of S100A9 and S100A12 Expression in Monocyte-Macrophage System in Hyperglycemic Conditions
Source: Front Immunol. 2020 Jun 2;11:1071. doi: 10.3389/fimmu.2020.01071 (PMC7280556; doi:10.3389/fimmu.2020.01071)
Supplement: Supplementary file 1 [file Data_Sheet_1.PDF]

TABLE OF CONTENTS

Supplementary Figure 1. S100A9 and S100A12 protein expression in primary human macrophages.. 2

Supplementary Figure 2. PBMCs from diabetic patients and healthy controls were analysed by flow cytometry. 3

Supplementary Figure 3. Classical, intermediate and non-classical monocytes subsets from T2D patients with microvascular complications 4

Supplementary Figure 4. Relation H3 occupancy and gene expression depends on promoter region in M1 macrophages. 5

Supplementary Figure 5. Modifications of histones at promoter regions in transient hyperglycemia. 6

Supplementary Figure 6. Scatter plot with bar presenting individual donor data for main Figure 8. .. 7

Supplementary Table 1. S100 gene family by affymetrix 8

Supplementary Table 2. Characteristics of individuals according to patient diagnosis........ 9

Supplementary Table 3. Correlation between *S100A9* and *S100A12* expression in monocytes........ 10

Supplementary Table 4. Clinical characteristics of the patients and control individuals. 11

Supplementary Table 5. Key Resource Table 12

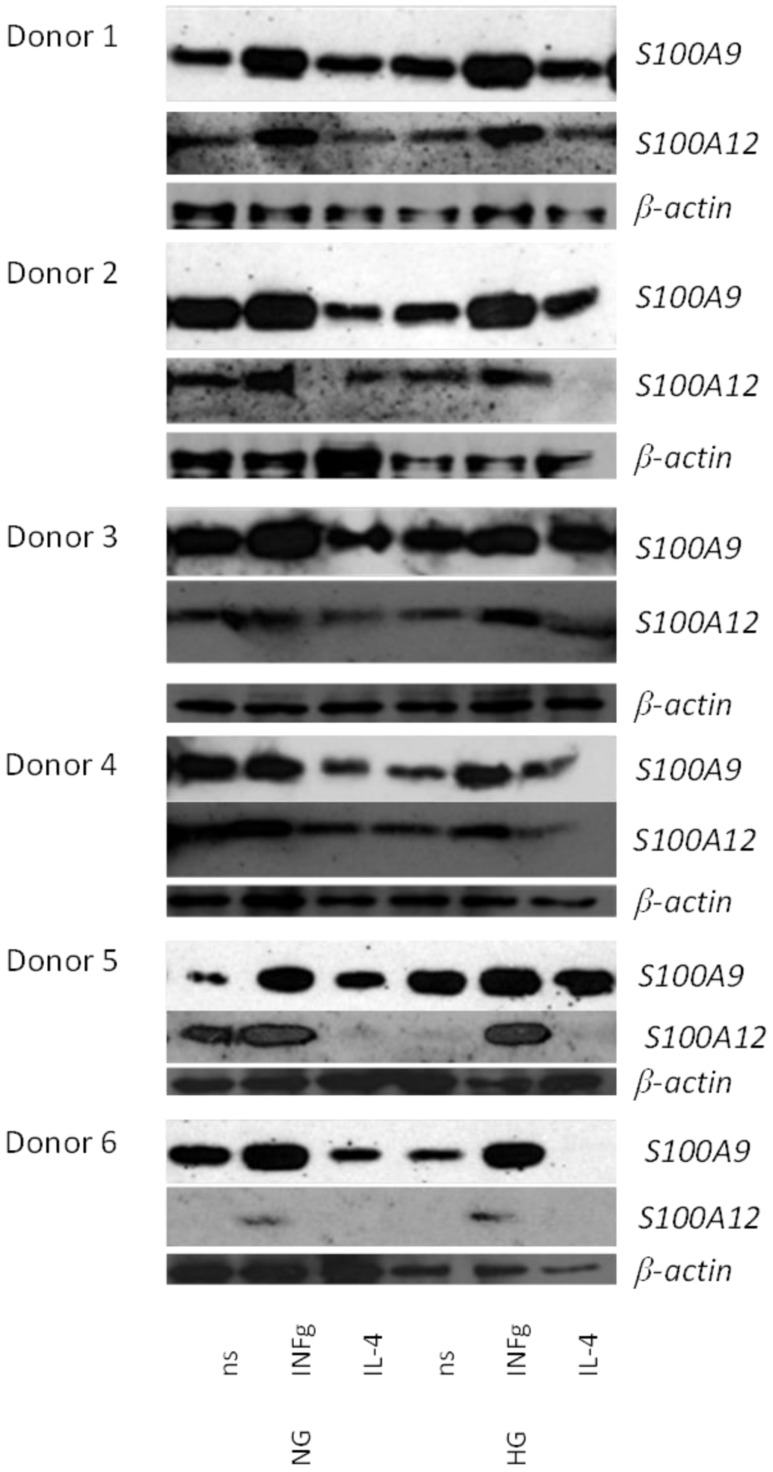

Supplementary Figure 1. S100A9 and S100A12 protein expression in primary human macrophages. Western Blot analysis of protein expression S100A9 and S100A12 (n=6) in M0, M1 and M2 macrophages cultured for 6 days cultured under normal (NG) and high glucose (HG) conditions.

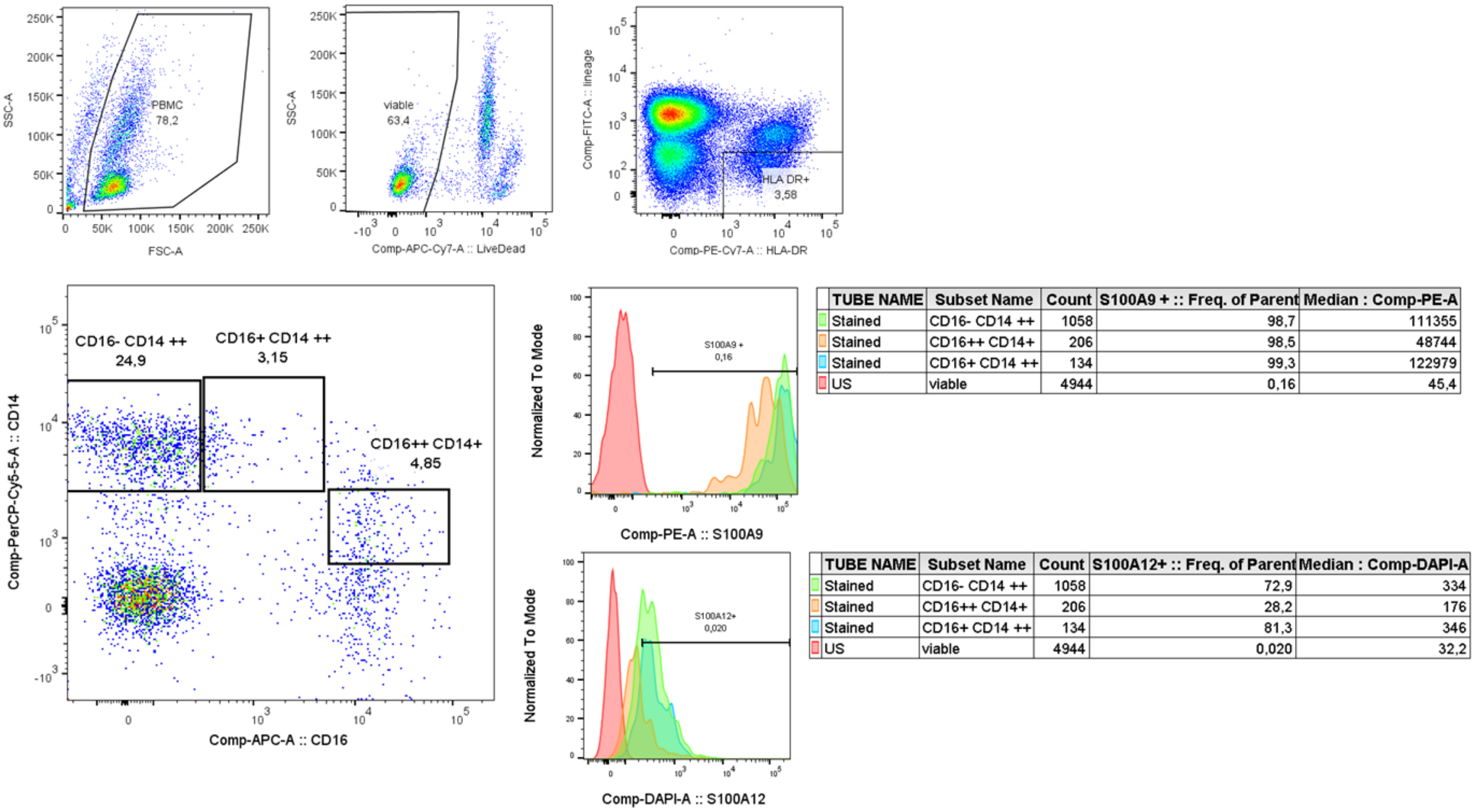

TUBE NAME	Subset Name	Count	S100A9 + :: Freq. of Parent	Median : Comp-PE-A
Stained	CD16- CD14 ++	1058	98,7	111355
Stained	CD16++ CD14+	206	98,5	48744
Stained	CD16+ CD14 ++	134	99,3	122979
US	viable	4944	0,16	45,4

TUBE NAME	Subset Name	Count	S100A12+ :: Freq. of Parent	Median : Comp-DAPI-A
Stained	CD16- CD14 ++	1058	72,9	334
Stained	CD16++ CD14+	206	28,2	176
Stained	CD16+ CD14 ++	134	81,3	346
US	viable	4944	0,020	32,2

Supplementary Figure 2. PBMCs from diabetic patients and healthy controls were analysed by flow cytometry. Cells positive for HLA-DR were selected and all cells expressing CD3, CD19 and CD56 were excluded. Using a scatter plot of CD16 versus CD14 monocyte population were separated into classical (CD14+CD16-), non-classical with low CD14 expression (CD14-CD16+) and intermediate (CD14+CD16+) monocytes. These populations were analysed for the expression S100A9 and S100A12.

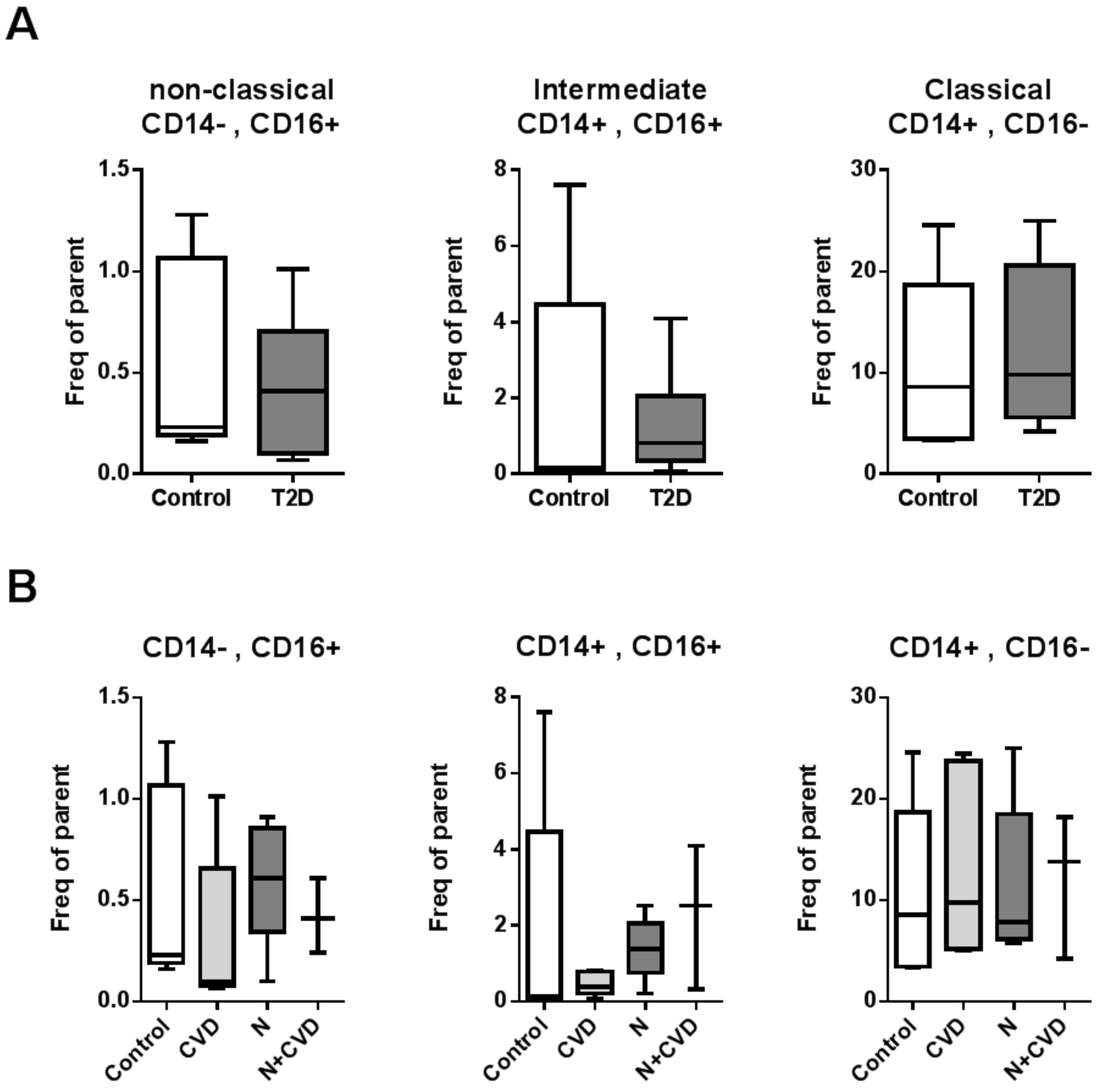

Supplementary Figure 3. Classical, intermediate and non-classical monocytes subsets from T2D patients with microvascular complications analysed based on CD14+ and CD16+ expression by FACs. **(**A) Monocyte subsets in total T2D group compared to healthy controls (B) Monocyte subsets in T2D patients with different vascular complications compared to healthy controls. Cardiovascular disease (CVD), nephropathy (N) and both, compared to healthy controls. Frequencies of parent (HLA-DR population) are plotted, Min to Max. Mann Whitney-U test (A) and One-way ANOVA (B) were used.

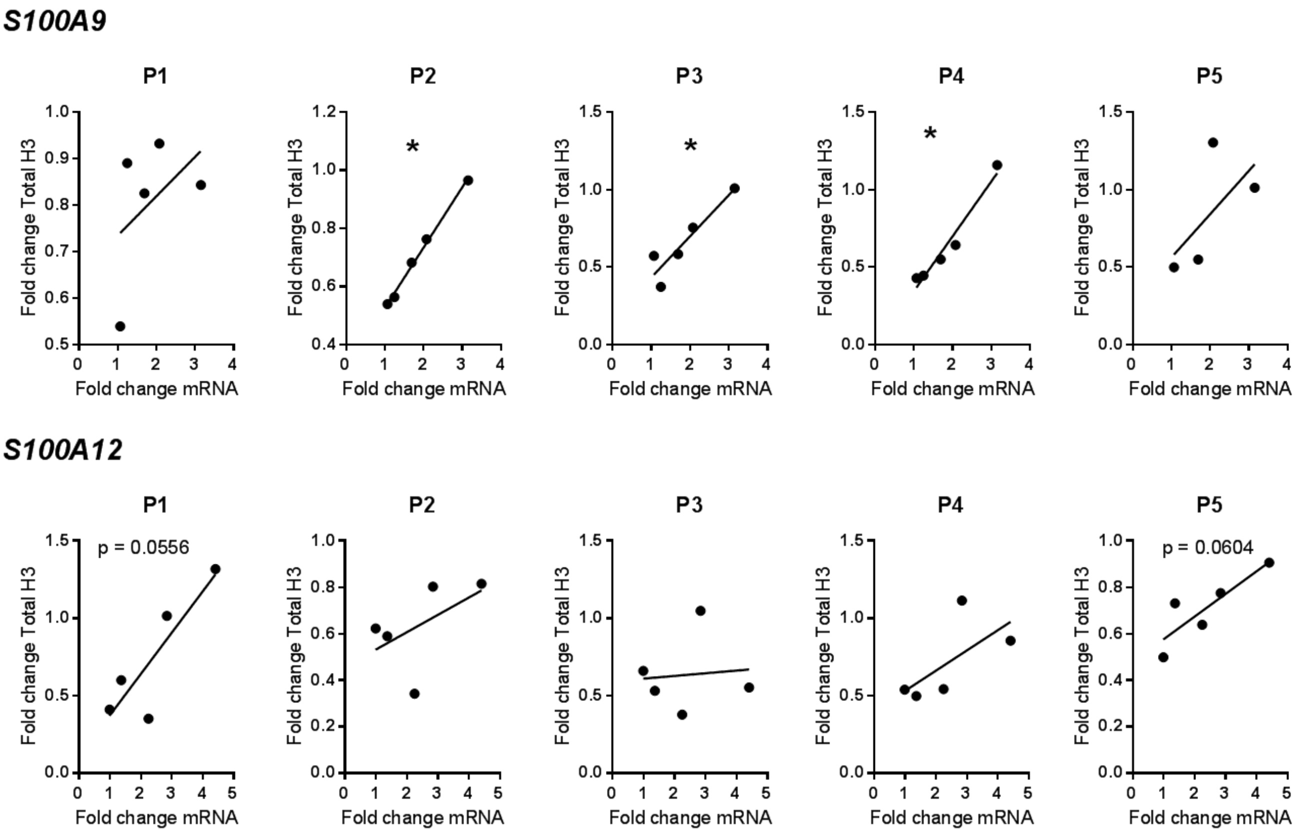

Supplementary Figure 4. Relation H3 occupancy and gene expression depends on promoter region in M1 macrophages. Linear regression of the histone mark association with fold change increase in gene expression (x-axis) with fold change increase of total H3 (y-axis) by culture under HG conditions. Correlation was shown for 5 single regions in promoters (P1 – P5) of *S100A9* (top) and *S100A12* (bottom). The correlation was considered to be statistically significant in case of *$p<0.05$ by linear regression analysis.

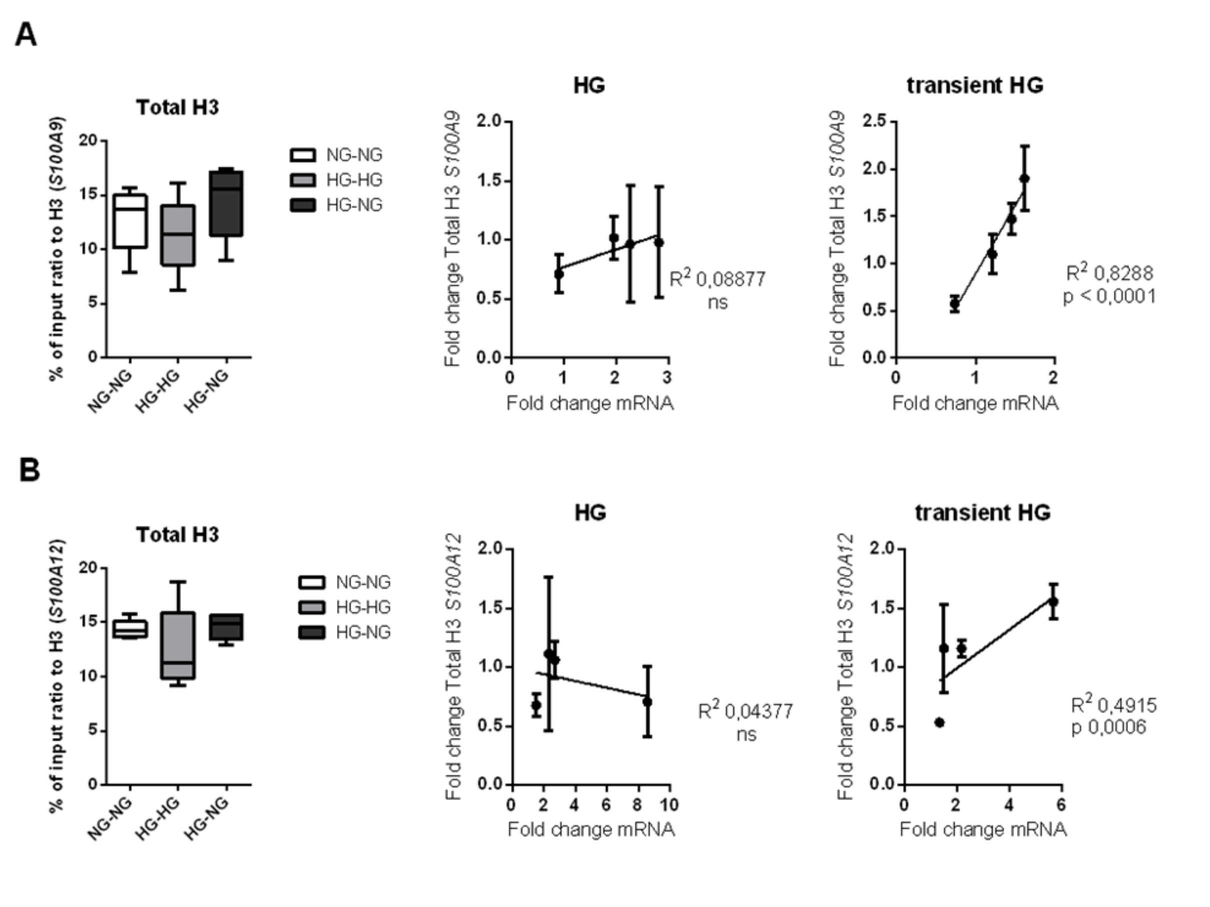

Supplementary Figure 5. Modifications of histones at promoter regions in transient hyperglycemia. Level of total H3 in *S100A9* (A) and *S100A12* (B) promoter regions, average of 5 regions, Min to Max plot and correlation of fold change increase in gene expression with increase of total H3 (n=4).

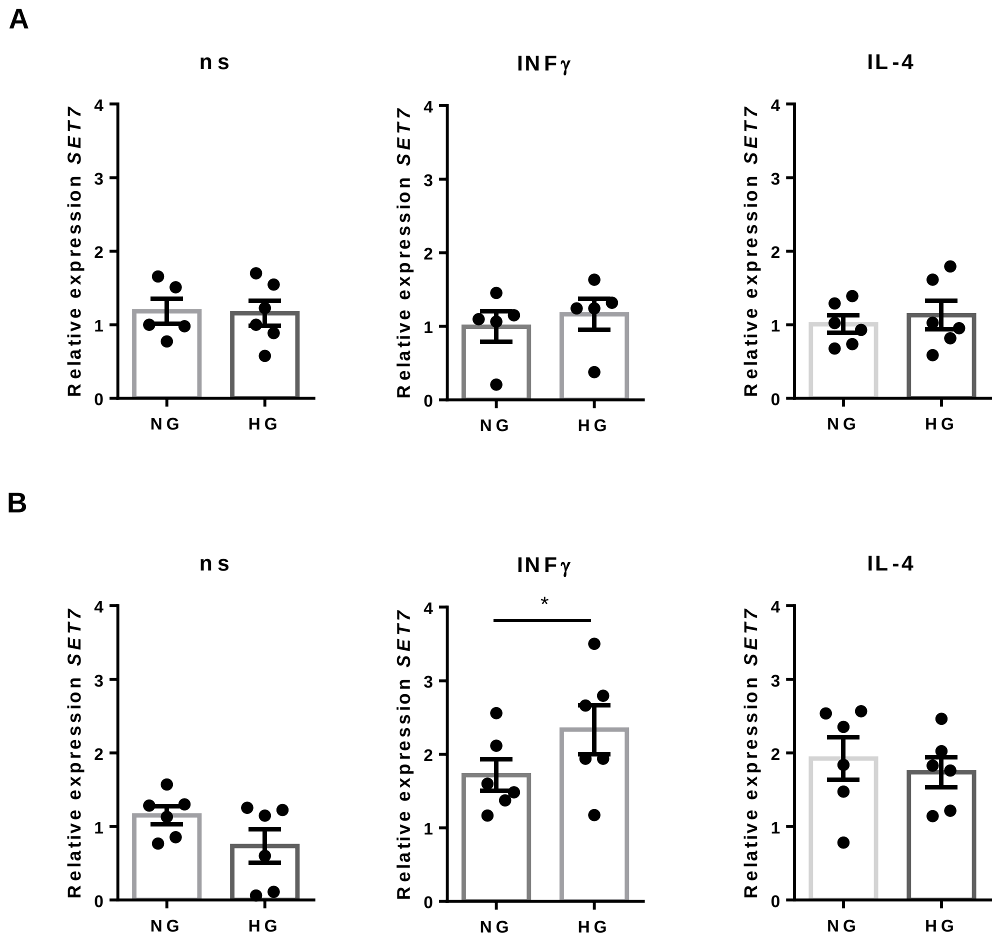

Supplementary Figure 6. Scatter plot with bar presenting individual donor data for main Figure 8. Relative expression of SET7 in primary human M0 ns, M1 IFNγ and M2 IL-4 macrophages after (A) 24h and (B) six days culture under NG and HG conditions. Data present mean ± SEM normalised to 18SrRNA levels. Statistical analysis was tested by Two-way ANOVA, n = 6.

Supplementary Table 1. S100 gene family by affymetrix

GeneSymbol	Fold change NS	Fold change IFNγ	Fold change IL-4	Adjusted p-Value for Estimate of NS	Adjusted p-Value for Estimate of IFNγ	Adjusted p-Value for Estimate of IL-4
S100A12	1,309409	4,411155	1,055020	0,368051	0,000035*	0,973707
S100A10	1,080588	1,287492	1,157048	0,478160	0,008734*	0,421370
S100Z	1,132830	1,658848	1,089954	0,674781	0,024781*	0,929235
S100A4	1,051011	1,970927	1,017411	0,894199	0,004853*	0,993473
S100A9	1,435917	2,130770	1,019306	0,073179	0,000687*	0,990445
S100A16	1,954496	1,004997	2,022121	0,020164*	0,990636	0,088720
S100A7L2	1,106120	1,023489	1,049944	0,029907*	0,677327	0,644794
S100A8	3,193302	1,865075	1,380401	0,003648*	0,066537	0,728683

*Every comparison is between cells cultured in high glucose conditions versus cells cultured in normal glucose conditions

Supplementary Table 2. Characteristics of individuals according to patient diagnosis

	T1D (n=19)	T2D (n=21)	Prediabetic (n=19)	Control (n=21)
Age (years)	50,2 ± 18,1	59,7 ± 6,1	59,1 ± 7,6	57,9 ± 7,4
Sex (women)	56%	48%	47,4%	57%
Diabetes duration (years)	31,1 ± 14,4	9,7 ± 6,0		
Size (cm)	172 ± 11	171 ± 10	171 ± 8	169 ± 10
Weight (kg)	79,8 ± 15,6	96,8 ± 19,6 *	97,8 ± 21,7 *	84,0 ± 18,2
BMI (kg/m2)	26,8 ± 4,3	31,4 ± 9,3	33,4 ± 7 *	29,2 ± 4,3
Smoking (%)	20,0	25,0	5,6	-
Retinopathy (%)	37,5 *	14,3	5,3	4,8
Polyneuropathy (%)	75,0 ***	76,2 ***	-	-
Fasting glucose (mg/dl)	177,2 ± 63,4 ***	167,0 ± 52,2 ***	106,7 ± 7,5 ***	90,2 ± 9,4
HbA1c	8,3 ± 1,6 ***	7,5 ± 1,2 ***	5,6 ± 0,4	5,5 ± 0,3
eGFR		95,6 ± 13,3	87,8 ± 11,9	92,9 ± 14,5
Urine Creatinine	91,9 ± 56,8	108,4 ± 63,3	130,5 ± 79,6	113,8 ± 57,2
Urine Albumin	39,9 ± 67,5	88,8 ± 137,8 *	13,9 ± 11,1	8,3 ± 4,0
U_ACR	16,0 ± 18,4	22,0 ± 27,4	2,9	
Nephropathy (%)	17,6 *	52,4 ***	11	-
NSS	4,0 ± 2,6	6,2 ± 2,5		
NDS	4,1 ± 3,0	4,5 ± 2,8		

Values represent mean ± SD or percentages (%). Control vs. other groups *p < 0.05, **p < 0.01 *** p < 0.001. BMI – Body Mass Index, eGFR - Estimated Glomerular Filtration Rate, ACR - albumin/creatinine ratio, NSS - Neuropathy Symptoms Score, NDS - Neuropathy Disability Score.

Supplementary Table 3. Correlation between S100A9 and S100A12 expression in monocytes from healthy controls, prediabetic individuals and diabetic patients, normalized to CD14 expression.

Patient PBMCs normalized to CD14+

	Control	*Prediabetic*	*T1D*	*T2D*
R^2	0,8091	0,1403	0,878	0,9778
p value	< 0,0001***	0,169	< 0,0001***	< 0,0001***

Supplementary Table 4. Clinical characteristics of the patients and control individuals.

Variable	Healthy controls (n=5)	T2D with complications (n=13)
Age (years)	54,0 ± 12,1	67,4 ± 10,8
Sex (%female)	100%	42%
BMI (kg/m2)	25,1 ± 2,4	31,5 ± 6,1
Waist circumference (cm)	85,4 ± 3,4	111,6 ± 14,2
Current smoke (%)	20%	16%
Hypertension	0%	83%
Cardiovascular disease (%)	0%	67%
Cholesterol (mg/dl) (mmol/l)	200,0 ± 46,0	196,2 ± 60,2
HDL (mg/dl) (mmol/l)	71,2 ± 29,6	44,5 ± 10,3
LDL (mg/dl) (mmol/l)	114,8 ± 26,4	107,8 ± 49,4
Triglycerides (mg/dl) (mmol/l)	70,6 ± 39,0	304,2 ±389,1
Glucose (mg/dl) (mmol/l)	89,8 ± 7,4	192,9 ± 101,4
HbA1c (%) (mmol/l)	5,4 ± 0,1	7,9 ± 1,4

Values represent mean ± SD or percentages (%). Control vs. other groups *$p < 0.05$, **$p < 0.01$ *** $p < 0.001$. BMI – Body Mass Index, HDL - High-density-lipoprotein, LDL – Low-density-lipoprotein, HbA1c - Hemoglobine A1c

Supplementary Table 5. Key Resource Table

**REAGENT or RESOURCE**	**CLONE**	**SOURCE**	**IDENTIFIER**
Antibodies			
CD16 APC	eBIOCB16 (CB16)	eBiosciences	Cat#17-0168-42
CD3 FITC	UCHT1	Biolegend	Cat#300406
CD19	HIB19	Biolegend	Cat#555412
CD56 FITC	5.1H11	Biolegend	Cat#362546
CD14 FITC	6103	Biolegend	Cat#11-0149-42
MHC II (HLA DR) PE/Cy7	L243	Biolegend	Cat#307616
Mouse monoclonal S100A9 antibody	MRP 1H9	Biolgend	Cat#350706
Mouse monoclonal S100A12 anitbody	161205	R&D Systems	Cat#1508105
Mouse monoclonal S100A8 antibody	749916	R&D Systems	Cat#MAB4570
Sheep polyclonal S100A9 antibody		R&D Systems	Cat#AF5578
Goat polyclonal S100A12 antibody		R&D Systems	Cat#AF1052
Rabbit polyclonal SET7/SET9 antibody		Cell Signaling	Cat#2813
Rabbit polyclonal Histone H3 (mono methyl K4) antibody		Abcam	Cat#8895
Rabbit polyclonal Trimethyl-Histone H3 (Lys4) antibody		Merck Millipore	Cat#07-473
Rabbit polyclonal Acetyl-Histone H3 Antibody		Merck Millipore	Cat#06-599
Histone H3 Rabbit mAb	D2B12	Cell Signaling	Cat#4620
Chemicals, Peptides, and Recombinant Proteins			
fixable viability dye		Invitrogen	Cat#65-0865-14
FcR Blocking Reagent human		Miltenyi Biotec	Cat#130-059-901
Recombinant Human M-CSF		Peprotech	Cat#300-25B
Recombinant Human Interferon gamma		Peprotech	Cat#300-02B
Recombinant Human IL-4		Peprotech	Cat#200-04B
D-(+)-Glucose solution		Merck Sigma	Cat#G8644
PFI-2 hydrochloride		MedChemExpress	Cat#HY-18627A
EPZ031686		MedChemExpress	Cat#HY-19324
WDR5 0103		R&D Systems	Cat#5323/10
Critical Commercial Assays			
E.Z.N.A. Total RNA Kit I		Omega bio-tek	Cat#R6834-02
QIAquick PCR Purification Kit		Qiagen	Cat#28104
SimpleChIP® Enzymatic Chromatin IP Kit		Cell Signaling Technology	Cat#9003
Human S100A9 DuoSet ELISA		R&D systems	Cat#DY5578
Human EN-RAGE DuoSet ELISA		R&D systems	Cat#DY1052-05
